# Circulating miR-16-5p, miR-92a-3p, and miR-451a in Plasma from Lung Cancer Patients: Potential Application in Early Detection and a Regulatory Role in Tumorigenesis Pathways

**DOI:** 10.3390/cancers12082071

**Published:** 2020-07-27

**Authors:** Patricia P. Reis, Sandra A. Drigo, Robson F. Carvalho, Rainer Marco Lopez Lapa, Tainara F. Felix, Devalben Patel, Dangxiao Cheng, Melania Pintilie, Geoffrey Liu, Ming-Sound Tsao

**Affiliations:** 1 Faculty of Medicine, São Paulo State University, UNESP, Botucatu, SP 18618-687, Brazil; sandra.drigo@gmail.com (S.A.D.); tainara.felix@unesp.br (T.F.F.); 2 Experimental Research Unity, São Paulo State University, UNESP, Botucatu, SP 18618-687, Brazil; 3 Department of Structural and Functional Biology, Institute of Biosciences, São Paulo State University, UNESP, Botucatu, SP 18618-689, Brazil; robson.carvalho@unesp.br; 4 Universidad Católica Los Ángeles de Chimbote, Instituto de Investigación, Chimbote 02800, Peru; reimco2@gmail.com; 5 Princess Margaret Cancer Centre, University Health Network, Toronto, ON M5G 1L7, Canada; Devalben.Patel@uhn.ca (D.P.); Dangxiao.Cheng@uhn.ca (D.C.); Melania.Pintilie@uhnresearch.ca (M.P.); Geoffrey.Liu@uhn.ca (G.L.); 6 Department of Medical Biophysics, University of Toronto, Toronto, ON M5G 1L7, Canada; 7 Department of Medical Oncology and Hematology, Princess Margaret Cancer Centre, Toronto, ON M5G 2C1, Canada; 8 Department of Laboratory Medicine and Pathobiology, University of Toronto, ON M5S 1A8, Canada; 9 Laboratory Medicine Program, University Health Network, Toronto, ON M5S 1A1, Canada

**Keywords:** lung cancer, lung adenocarcinoma, lung squamous cell carcinoma, microRNAs, plasma, liquid biopsy, biomarkers, lung tumorigenesis, pathways

## Abstract

Background: Micro(mi)RNAs, potent gene expression regulators associated with tumorigenesis, are stable, abundant circulating molecules, and detectable in plasma. Thus, miRNAs could potentially be useful in early lung cancer detection. We aimed to identify circulating miRNA signatures in plasma from patients with lung adenocarcinoma (LUAD) and lung squamous cell carcinoma (LUSC), and to verify whether miRNAs regulate lung oncogenesis pathways. Methods: RNA isolated from 139 plasma samples (40 LUAD, 38 LUSC; 61 healthy/non-diseased individuals) were divided into discovery (38 patients; 21 controls for expression quantification using an 800-miRNA panel; Nanostring nCounter^®^) and validation (40 patients; 40 controls; TaqMan^®^ RT-qPCR) cohorts. Elastic net, Maximizing-R-Square Analysis (MARSA), and C-Statistics were applied for miRNA signature identification. Results: When compared to healthy individuals, 580 of 606 deregulated miRNAs in LUAD and 221 of 226 deregulated miRNAs in LUSC had significantly increased levels. Among the 10 most significantly overexpressed miRNAs, 6 were common to patients with LUAD and LUSC. Further analysis identified three signatures composed of 12 miRNAs. Signatures included miRNAs commonly overexpressed in patient plasma. Enriched pathways included target genes modulated by three miRNAs in the C-Statistics signature: miR-16-5p, miR-92a-3p, and miR-451a. Conclusions: The 3-miRNA signature (miR-16-5p, miR-92a-3p, miR-451a) had high specificity (100%) and sensitivity (84%) to predict cancer (LUAD and LUSC). These miRNAs are predicted to modulate genes and pathways with known roles in lung tumorigenesis, including *EGFR*, *K-RAS*, and *PI3K/AKT* signaling, suggesting that the 3-miRNA signature is biologically relevant in adenocarcinoma and squamous cell carcinoma of the lung.

## 1. Introduction

Lung cancer is the most frequent type of cancer in both sexes worldwide, with over 2 million newly diagnosed cases every year. It is the leading cause of cancer death, with over 1.8 million deaths per year. Lung cancer incidence and mortality are expected to rise to over 3.6 million new cases and over 3 million deaths/year by 2040. Currently, the 5 year survival rate is only ~15–20% [1]; the Surveillance, Epidemiology and End Results (SEER) database indicates that the mortality rate by lung cancer has remained unaltered in the past 5 years [2]. Despite advances in its treatment, lung cancer morbidity and mortality remain high, mainly due to diagnosis in advanced disease stages. Therefore, there is an urgent need for improved early lung cancer detection to achieve cure.

miRNAs are small, non-coding RNAs predicted to regulate over 50% of human genes [3]. In cancer, miRNAs act as oncogenes or tumor suppressor genes, leading to the deregulation of a large number of genes associated with oncogenesis [4,5]. miRNAs are stable molecules and can be detected in clinically relevant samples, including tissues and body fluids. In cancer patients, miRNAs are abundant as circulating molecules: actively secreted by tumor cells into exosomes, passively released in the extracellular space bound to the Argonaut 2 protein (Ago2), or as free molecules derived from apoptotic or necrotic tumor cells [6,7]. Tumor cells have an increased release of miRNAs when compared to normal tissues, with altered circulating expression levels of miRNAs from cancer patients, possibly reflecting tumor miRNA expression. Indeed, the detection of circulating miRNAs in the peripheral blood of cancer patients may provide insights into the tumorigenesis process, including tumor development and signaling mechanisms of metastasis [5,8].

Circulating miRNAs have been reported in lung cancer [9,10] and are considered to have promising clinical value as blood-based biomarkers [5]. Previous studies have investigated circulating miRNAs as potential biomarkers of early diagnosis in lung cancer [11,12,13,14,15,16,17,18,19,20,21,22,23,24,25,26,27,28,29,30]. Selected literature studies for early detection of lung cancer are summarized in Table S1. Despite the contributions of these studies, there are limitations with most studies assessing a limited number of miRNAs and lacking validation, thus limiting the identification of clinically useful signatures.

The development of strategies for early detection of lung cancer is still a growing field, and essential for improving patient survival. Therefore, we aimed: (a) to identify miRNA signatures in plasma from patients with LUAD or LUSC, and (b) to investigate the association of circulating miRNAs and their target genes with lung tumorigenesis pathways.

## 2. Results

### 2.1. Higher Levels of Plasma miRNAs are Found in LUAD and LUSC Cases Versus Controls

**Figure 1:**
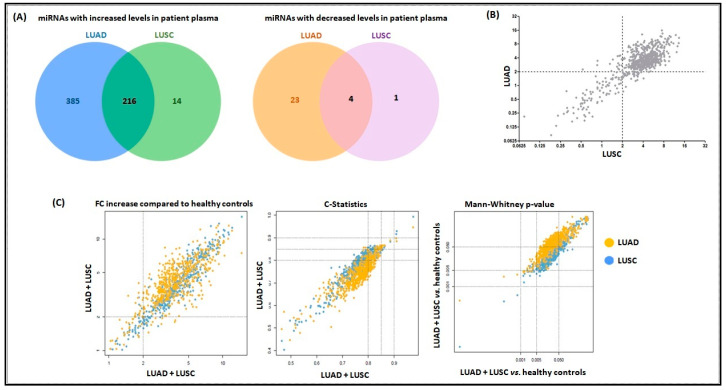
miRNAs have commonly deregulated levels and are correlated in patient plasma. Panel (**A**) shows the Venn diagram with the distribution of miRNAs that showed significantly increased or decreased levels in plasma from LUAD and LUSC patients, respectively. Most miRNAs have similar levels of deregulation in patient plasma independent of tumor histology. LUAD: lung adenocarcinoma; LUSC: lung squamous cell carcinoma. Panel (**B**) shows the correlated expression for the 800 miRNAs in the Nanostring nCounter panel (Spearman test, r = 0.67, *p* < 0.0001). Note that most miRNAs have FC ≥ 2.0 in plasma from patients with both tumor histologies. Panel (**C**) are the scatter plots to depict a high correlation between overexpressed miRNAs in plasma from patients with LUAD and LUSC.

We detected 628 miRNAs with statistically significantly altered levels (FC ≥ 2 and *p* < 0.05) in plasma of patients with LUAD and 235 miRNAs in plasma of patients with LUSC (Tables S2 and S3, respectively), when compared to reference control plasma samples. Interestingly, most miRNAs had significantly increased levels in patient plasma compared to healthy individuals: in LUAD, 601 miRNAs were overexpressed and 27 were underexpressed; in plasma from LUSC patients, 230 miRNAs were overexpressed, and five miRNAs were underexpressed. Importantly, 216 overexpressed miRNAs and four underexpressed miRNAs were found across both tumor histologies in common (Table S4, Figure 1A). A positive correlation (r = 0.67, *p* < 0.0001) was observed between miRNA expression levels in the plasma from LUAD and LUSC patients, for all 800 miRNAs assessed in the Nanostring nCounter platform, regardless of FC values and statistical significance. This verifies that plasma miRNA levels were similar for patients with both histological tumor subtypes (Figure 1B). Notably, overexpressed miRNAs in the plasma of patients with LUAD and LUSC were highly correlated, indicating a commonality between the histological subtypes regarding miRNA expression in patient plasma (Figure 1C).

Among the miRNAs overexpressed in patient plasma, miR-16-5p was the most significantly altered in all comparisons: cancer cases vs. healthy controls, LUAD vs. healthy controls, and LUSC vs. healthy controls. Additionally, among the top 10 most significantly altered miRNAs in patient plasma, 6 miRNAs (miR-16-5p, miR-451a, miR-92a-3p, miR-25-3p, miR-1285-5p, and miR-155-5p) were detected with common high levels in plasma from patients with both tumor histologies (Table 1).

### 2.2. miRNA Signatures Have Potential Application in Early Detection of LUAD and LUSC

**Figure 2:**
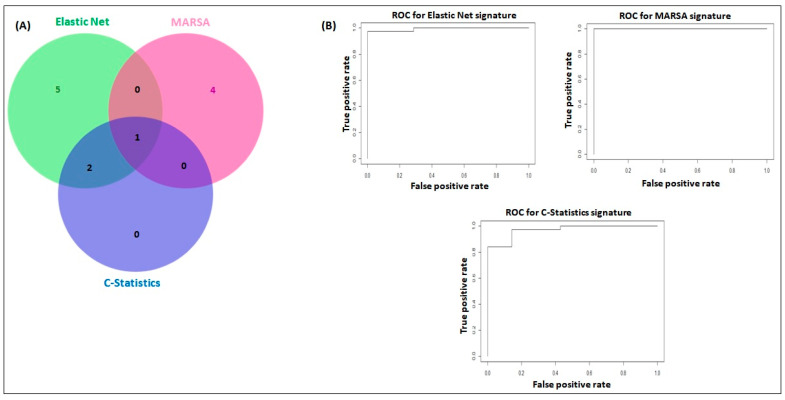
Plasma miRNA signatures. Panel (**A**) is the Venn diagram showing the identified signatures by Elastic net (eight miRNAs: miR-16-5p, miR-92a, miR-451a, miR-106b-5p, miR-155-5p, miR-217, miR-1285-3p, miR-1285-5p), MARSA (five miRNAs: miR-16-5p, miR-148b-3p, miR-378e, miR-484, miR-664a-3p), and C-Statistics (three miRNAs: miR-16-5p, miR-92a, miR-451a). The miRNAs miR-16-5p, miR-92a, and miR-451a were shared among Elastic net and C-Statistics signatures. miR-16-5p was the only miRNA shared by all signatures. Panel (**B**) shows the ROC, Sensitivity and Specificity data for identified miRNA signatures: Elastic net, MARSA, and C-Statistics.

To identify plasma miRNA signatures associated with lung cancer patients, the application of three different statistical approaches (Elastic Net, MARSA, and C-Statistics) allowed us to identify three distinct signatures that involved 12 individual miRNAs: miR-16-5p, miR-92a, miR-451a, miR-106b-5p, miR-155-5p, miR-217, miR-1285-3p, miR-1285-5p, miR-148b-3p, miR-378e, miR-484, and miR-664a-3p (Table 2). Five miRNAs were exclusive to the Elastic Net-derived signature, four miRNAs were exclusive to the MARSA signature, while two miRNAs were shared between Elastic Net and C-statistics. All three signatures (Figure 2A) shared one miRNA (miR-16-5p). Notably, 5 of the 12 signature miRNAs were among the top 10 most significantly overexpressed miRNAs in plasma from LUAD and LUSC cases: miR-16-5p, miR-451a, miR-92a-3p, miR-1285-5p, and miR-155-5p. For the Elastic net signature (eight miRNAs), risk scores varied between 3.5–162.5, with a median value of 19.5. In the Elastic net signature, specificity was 100%, while sensitivity was 97% (38/40 cases detected). When applying MARSA, we identified a five-miRNA signature, with miR-16-5p being shared between the Elastic net and MARSA signatures. MARSA showed median risk scores of 10.4 and 2.1 for cancer patients and controls, respectively, with SD = 11.8. MARSA specificity and sensitivity were 100%. In the third method, we combined all miRNAs with C-Statistics ≥ 0.9, which resulted in a C-Statistics signature composed of three miRNAs: miR-16-5p, miR-92a, and miR-45. Interestingly, these miRNAs were also identified in the Elastic net signature. The C-Statistics signature showed median values for cancer cases and controls of 23 and 4.6, respectively, with specificity of 100% and sensitivity of 84% (34/40 cancer patients were correctly identified). The ROC curves for all three signatures are shown in Figure 2B. Additionally, we identified a high correlation between the continuous variables of the signatures, with Spearman correlations of 0.87, 0.89, and 0.98 for Elastic net, MARSA, and C-Statistics, respectively.

### 2.3. miRNA Signatures and Individual miRNA Levels Were Verified in a Separate Set of Plasma Samples

**Figure 3:**
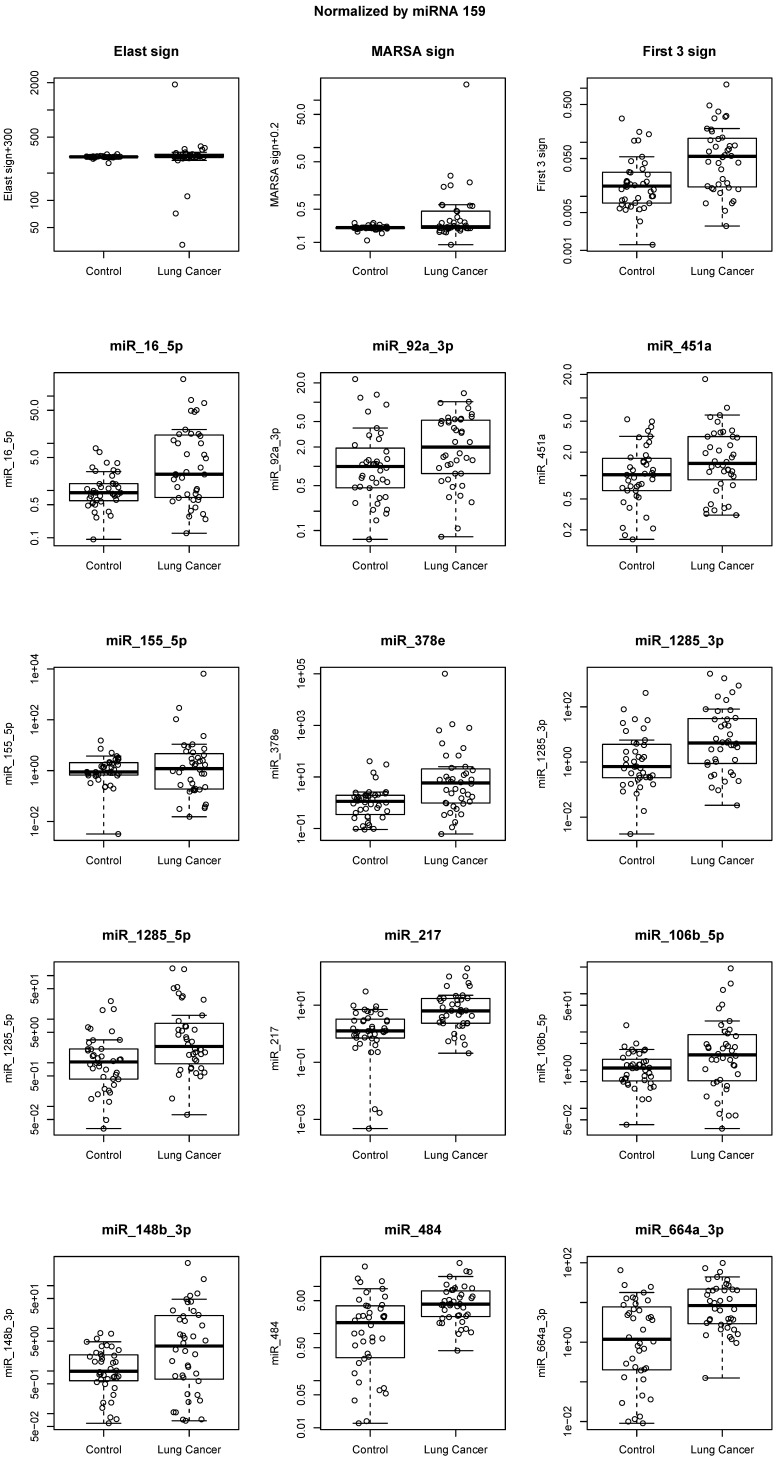
Validation data showing miRNA levels in the signatures (Elastic net, MARSA, and C-Statistics) and individual miRNA levels for the first three C-Statistics miRNAs: miR-16-5p, miR-92a-3p, and miR-451a, as well as the remaining miRNAs (miR-155-5p, miR-378e, miR-1285-3p, miR-1285-5p, miR-217, miR-106b-5p, miR-148b-3p, miR-484, and miR-664a-3p) that are included in the identified signatures. *Y*-axis represents miRNA levels determined by the Delta Ct method [31]. Data were normalized against the ath-mir-159a exogenous control. In addition, we show that data normalization against a stably expressed endogenous control (miR-93) resulted in similar miRNA levels for all signatures and individual miRNAs, demonstrating consistency of identified miRNA levels across all samples (data are shown in Figure S1).

Next, we assessed the expression of the 12 miRNAs as signatures, as well as individually in a second sample set, in order to validate our result. Validation experiments used a different platform: quantitative real-time RT-PCR (RT-qPCR) with TaqMan^®^ miRNA assays. The three signatures confirmed the Nanostring results (discovery set), with all signature miRNAs showing higher levels in plasma from cases than controls. The C-Statistics signature had the best performance (*p* = 0.00019, C-Statistics = 0.727) (validation results, Table 3, Figure 3). Expression levels for the 12 miRNAs were also analyzed individually, confirming 11 of 12 miRNAs that were identified in our discovery set, including the entirety of the C-Statistics signature miRNAs. The miR-155-5p was the only one that did not show concordant levels between discovery and validation sets (Table 3, Figure 3).

### 2.4. miR-16-5p, miR-92a-3p and miR-451a Target Genes are Associated with Lung Tumorigenesis Pathways

We next sought to identify molecular pathways regulated by genes targeted by miR-16-5p, miR-92a, and miR-451a, which formed the C-Statistics signature but also partly shared with the Elastic Net and/or MARSA signatures. These three miRNAs were selected for pathway analysis, since they had the best statistical performance. In this analysis, we first predicted gene targets for these three miRNAs using miRDIP [32], which resulted in 4094 predicted miRNA targets. Further, miRNA-target gene interactions were searched in mirTarBase to confirm experimentally-validated miRNA-targets; this analysis identified 95 genes that had confirmed regulation by the C-Statistics miRNAs. We then verified whether these 95 genes were expressed in normal lung tissues using data from the Genotype-Tissue Expression (GTEx) database [33] in a set of 383 normal lung tissues; this analysis showed that 84 of 95 genes were expressed in normal lung. These data, along with the bioinformatic analyses study design are shown in Figure S2. Interestingly, among the genes regulated by these miRNAs, *BCL2* was identified as a common target of miR-16-5p and miR-451a (Table S5).

**Figure 4:**
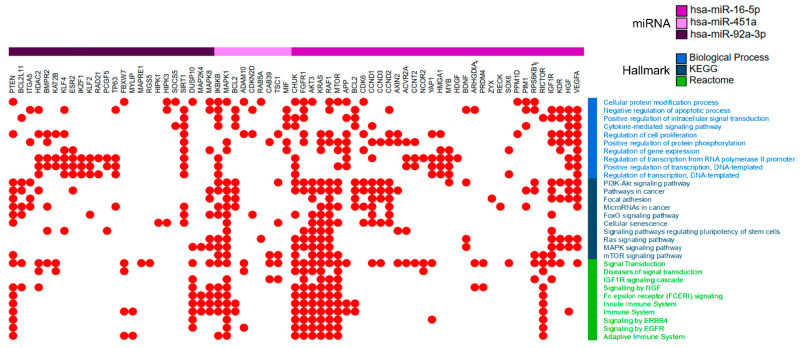
Pathways regulated by genes targeted by miRNAs. Each miRNA is shown associated with their specific target genes, in the above color bar: miR-16-5p (purple color), miR-92a-3p (violet), and miR-451a (light pink). Pathways shown in the figure were selected among the top 10 most significant, enriched pathways (*q*-value < 0.01, FDR < 0.05) and that included at least 10 participating genes in each pathway. The red dots indicate the respective pathway(s) in which genes are involved. Biological process (light blue), KEGG (dark blue) and Reactome (green) hallmarks are in the right panel

We then verified the expression of these 84 genes (expressed in normal lung tissues) in the LUAD and LUSC-TCGA (The Cancer Genome Atlas) datasets. When compared to GTEx normal lung tissues (*n* = 383) using GEPIA (see Methods), integrative gene expression analysis in the TCGA tumor datasets (*n* = 549 LUAD and 504 LUSC) showed that a number of these genes was deregulated (under- or overexpressed) in tumors (shown in Table S6). Pathway enrichment analysis (Enrichr tool) identified statistically significantly enriched pathways associated with lung cancer. Table S7 shows the gene ontology (GO) biological processes and KEGG and Reactome classification of our data. Notably, EGFR, PI3K/AKT, MAPK1, FGFR1, RAF1, RAS, MAPK, and mTOR signaling were among the enriched pathways regulated by genes targeted by miRNAs: miR-16-5p, miR-92a, and miR-451a (Figure 4).

## 3. Discussion

Circulating miRNAs have been suggested as biomarkers to improve the diagnosis or early detection of lung cancer [11,12,13,14,15,16,17,18,19,20,21,22,23,24,25,26,27,28,29,30]. Here, we report that several miRNAs were detected at significantly higher levels in patient plasma with a large overlap of altered miRNAs between LUAD and LUSC. This lung tissue specificity was seen in another study that reported a 20-miRNAserum signature in LUAD, LUSC, and large cell carcinoma [26].

In our data, few miRNAs had lower abundance in plasma from cancer patients when compared to healthy individuals. Among miRNAs with significantly high levels, 12 exclusive miRNAs made up three distinct signatures with high sensitivity and specificity for detection of LUAD and/or LUSC. Notably, the 3-miRNA C-Statistics signature (miR-16-5p, miR-92a, and miR-451a) is included among miRNAs that compose a 24-miRNA classifier (MSC) described by Sozzi et al. [22], which is one of the largest studies investigating a high-risk screening population. Despite differences in study design, goals, samples, and patients, these three miRNAs were found within the MSC classifier. This finding contrasts with other studies that observed low degrees of overlap between signatures [34]. Our 3-miRNA signature (miR-16-5p, miR-92a-3p, and miR-451a) may be robust for lung cancer early detection, since it was distinctly found in lung cancer patients only, which were mostly of Stage I (62%; 48/78).

Published studies have focused on validating signatures or optimizing methodologies for the development of lung cancer diagnostic tests [35,36,37]. Other studies have focused on identifying miRNA classifiers, signatures, or individual circulating miRNAs for early detection or to supplement lung cancer diagnostic strategies. A 20-miRNA signature showed 89% sensitivity and 95% specificity [26]. Another study reported a 10-miRNA serum signature useful for lung cancer diagnosis in 400 LUAD, LUSC and large cell carcinoma cases and 220 controls. Boeri et al. [14] identified 16 ratios among 13 miRNAs from a training group of 19 patients and five pooled serum samples from healthy individuals, and validated their data in patients and pooled controls. Later, this same group generated a modified classifier of 18 miRNAs, in 27 patient samples, and tested it on 69 cases and 870 controls.

The above studies used amplification-based methods; our study used the Nanostring nCounter^®^ technology. The advantages of using this platform include no requirement of high DNA or RNA sample quantity or quality for analysis. The method is based on hybridization and signal counting, directly quantifying the molecules of interest and thus avoiding the bias introduced by amplification in PCR-based assays [38]. A previous study by our group showed that Nanostring technology was able to accurately detect changes in the expression of 20 genes in paired frozen and formalin-fixed paraffin-embedded (FFPE) oral carcinoma tissues; the same result was not observed using quantitative real-time PCR [39]. Nanostring has been shown to have high sensitivity and specificity and assay reproducibility, which are critical features of clinical utility [40]. More recently, this technology was used to develop a molecular assay that determines the risk of recurrence in oral cancer [41] and triple-negative breast cancer patients [42]. Our results, along with others, suggest that the Nanostring assay is useful for molecular testing development across multiple tumor types, including early detection of lung cancer.

In the literature, identified miRNA signatures or individual miRNAs should be considered with caution for those reports that do not specify the miRNA strand or do not provide the original, raw data. Besides, detection of circulating miRNAs with a potential clinical application can be affected by different factors, including variations due to the use of amplification to detect and quantify changes in miRNA abundance, and the use of endogenous or exogenous miRNAs for normalization.

MiR-16-5p, which is part of our C-Statistics signature, has been widely used as an endogenous control for data normalization. However, this miRNA was found with significantly deregulated levels in cancer; miR-16 overexpression was associated with osteoclast differentiation and bone metastasis [43], and variable, aberrant miR-6 levels were associated with breast cancer in patients with and without lymph node metastasis [44]. These findings demonstrate a variable expression pattern of miR-16, as well as other miRNAs, in different tumors, and indicate that a standardized reference for data normalization, including exogenous spike in controls, should be established for studies assessing miRNA expression, especially for circulating molecules in plasma or serum from cancer patients (Reviewed in [45]).

To assess the biological significance of extracellular or free circulating miRNAs in tumorigenesis, we showed that the target genes of miR-16-5p, miR-92a-3p, and miR-451a are expressed in normal lung tissue. These genes are involved in the regulation of key signal transduction pathways in lung cancer. Among identified pathways, we highlight the EGFR, FGFR1, KRAS, and PI3K-AKT signaling, and other well-known lung cancer pathways, such as MAPK and mTOR. Recently, in vitro and in vivo xenograft models showed that FGFR1 cooperates with EGFR in LUAD and that this cooperative interaction could be used to justify dual receptor inhibition in EGFR-activated tumors with up-regulated FGFR1 expression [46]. A study by Lu et al. [47] showed that miR-92a targets and inhibits *PTEN* in lung cancer cells, leading to PI3K/AKT signaling activation associated with tumorigenesis. Targeting specific mutations of KRAS is now possible, as is targeting the proteins downstream of KRAS, such as Rho [48]. Changes in various miRNAs were reported in plasma from patients with EGFR-mutated tumors, with or without resistance to tyrosine kinase inhibitors; however, miRNAs identified in this study do not overlap with our signature miRNAs [49].

To the best of our knowledge, only two studies that investigated circulating miRNAs in lung cancer patients performed extensive bioinformatic analysis for pathway identification [14,50]. In contrast to these, our study provides a comprehensive analysis and biological interpretation of plasma miRNA data. In our study, we showed: (a) prediction of miRNA target genes, (b) identification of target genes expressed in normal lung tissue in a large RNA-Seq dataset, (c) verification of gene expression patterns in a large number of lung cancer samples (TCGA), normalized against a large collection of normal lung tissues (GTEx dataset), and (d) correlated miRNAs, target genes, and pathways. In addition, we provide access to the original data and complete gene lists.

In our study, we investigated 800 miRNAs in a discovery set of 38 patients and 21 controls; we verified overexpression of 11 of 12 miRNAs, and then validated a 3-miRNA signature in a separate set of 40 patients and 40 controls. In the literature, only 3 of 21 published studies investigated a similarly large number of miRNAs (detailed in Table S1): Wozniak et al. [28] investigated plasma from 100 patients and 100 controls from Russia, but did not report an independent validation set. Halvorsen et al. [17], analyzed serum samples from 38 patients and 16 controls (discovery set), and validated 6 of 7 miRNAs in 51 patients and 107 screening trial individuals, also from Europe. Tai et al. [26] analyzed serum samples from 143 patients and 49 controls, and validated their data in 110 patients, 52 controls, and 47 patients with benign lung diseases, from Asia. In conjunction with these other studies, our data contributes by identifying a stable, consistent 3-miRNA plasma signature in patients with LUAD and LUSC in a fourth distinct population, a North American sample. Nevertheless, testing the diagnostic performance of miRNA signatures for early detection of lung cancer is still required for clinical application, using large cohorts of patients and controls, ideally from geographically distinct populations.

## 4. Material and Methods

### 4.1. Study Population

The study protocol was approved by the University Health Network Research Ethics Board (REB#: 06-639). Plasma samples were collected from treatment-naïve patients (cases) with a confirmed diagnosis of lung adenocarcinoma (LUAD) or squamous cell carcinoma (LUSC). Exclusion criteria and inclusion were applied, as outlined in Figure S3. Lung cancer patients were selected based on availability of plasma samples and complete demographic, clinical, and histopathological information. Age- and sex- matched reference plasma samples (controls) were obtained from healthy individuals who were participating in the Lusi Wong Early Detection of Lung Cancer program; these controls had received low-dose computerized tomography screening studies at the Princess Margaret Cancer Centre, University Health Network, Toronto, ON, Canada [51,52]. The distribution of stage at diagnosis for cases was chosen to reflect the distribution observed in our underlying lung cancer screening program; 87% were early stage, of which 71% were Stage I. Clinico-demographic and pathological information was recorded and is shown in Table S8.

Sample size was determined by cost, feasibility and availability of samples. Cases were 78 lung cancer patients randomly selected and distributed into discovery (*n* = 38; 22 LUAD and 16 LUSC) and validation sets (*n* = 40; 18 LUAD and 22 LUSC), respectively, with 61 reference control samples (21 in the discovery set and 40 in the validation set). Given that baseline characteristics of LUAD and LUSC were different, matching was distribution-based rather than individual-based. During enrollment of cases and controls, peripheral blood was collected in EDTA vacuum tubes, and plasma was immediately separated by two centrifugation steps at 4000 rpm at 4 °C, for 10 min each, to ensure that cell-free plasma was obtained, following by storage at −80 °C.

The study design and main results are summarized in Figure S3**.**

### 4.2. RNA Extraction

Two hundred microliters (200 µL) of plasma were used for RNA extraction with the miRNeasy Serum/Plasma Kit (Qiagen, Toronto, ON, Canada), following the manufacturer’s protocol. This method allows purification of total cell-free RNA. During plasma extraction, 5 µL of a 200 pM solution of two exogenous synthetic spike-in controls (ath-miR-159a and cel-miR-248, sequences described in Table S9; Integrated DNA Technologies -IDT, Coralville, IA, USA), were added after sample lysis, according to the Nanostring protocol. These exogenous controls were used for data normalization as an efficient strategy to reduce miRNA expression bias, due to differences in RNA yield, a critical aspect of plasma samples, which can have significant differences in circulating RNA yields between individuals [53,54]. Following extraction, RNA quantification was performed using Nanodrop 1000 (Thermo Fisher Scientific, Waltham, MA, USA), and samples were stored at −80 °C until miRNA expression analysis.

### 4.3. Quantitative MiRNA Expression Analysis by Nanostring NCounter^®^ Assay

The miRNA expression data in the discovery set was generated using the Human v3 miRNA Expression panel (Nanostring nCounter^®^ assay) (Nanostring Technologies, Seattle, WA, USA), containing 800 miRNAs with 100% coverage of the miRBase high confidence [55,56,57] and clinically relevant miRNAs [58,59]. The Nanostring nCounter^®^ assay used 200 ng of RNA from each sample. This assay was performed at the Princess Margaret Genomics Centre (https://www.pmgenomics.ca/pmgenomics/), according to the manufacturer’s protocol, and as described in previous studies [41]. The nSolver™ Analysis Software (www.nanostring.com/nsolver) was used for global normalization of miRNA expression data against positive and negative controls, exogenous and stable endogenous controls, and to calculate fold change (FC) and associated *p*-values for deregulated miRNAs in patient plasma compared to reference, healthy controls. Original, raw Nanostring data are available in Gene Expression Omnibus under accession number GSE152702.

### 4.4. Validation of MiRNA Expression by TaqMan^®^ Quantitative Real-Time PCR

Validation analysis was performed in a blinded fashion, with control and patient samples blinded to the data analyst (PPR) until all analyses were completed. Significantly deregulated miRNAs included in the three identified signatures were validated by quantitative real-time PCR using the TaqMan^®^ Advanced miRNA assays (Thermo Fisher, Foster City, CA, USA) on the 7900 Sequence Detection System (Thermo Fisher Scientific), which allows highly sensitive and specific amplification of miRNAs. Primer sequences are shown in Table S10. Protocols followed the manufacturer’s recommendations. Briefly, RNA (5 ng/µL) was used for poly (A) tailing reaction under thermal-cycling conditions (37 °C for 45 min for polyadenylation, 65 °C for 10 min to stop the reaction and a hold step at 4 °C), followed by adaptor ligation (16 °C for 60 min for ligation and a hold step at 4 °C). The third step is the reverse transcription (RT) using a universal miRNA RT primer that allows for cDNA synthesis and amplification of any target miRNA with the following conditions (42 °C for 15 min, 85 °C for 5 min and 4 °C hold). RT products were used for TaqMan quantitative PCR, as follows: a 1:10 dilution of each RT product was prepared by adding 5 µL of miR-Amp RT products to 45 µL ddH2O. Quantitative PCR was performed using the TaqMan^®^ Advanced miRNA assay and TaqMan^®^ Advanced Master Mix. Amplification was performed under the following cycling conditions: 1 cycle at 95 °C for 20 s for enzyme activation and 40 cycles at 95 °C for 3 s for denaturation and 60 °C for 30 s. for annealing and extension. All reactions included technical duplicates. miRNA expression data were calculated using the Delta Delta Ct method [31]. To date, there is no consensus in the literature about an ideal normalization method in the analysis of circulating miRNAs in plasma. The qRT-PCR data was normalized using the exogenous ath-miR-159 or the endogenous miR-93 as controls, and the results were compared. However, since normalization using exogenous controls was demonstrated to lower intra-sample variability, and to offer a two-fold lower inter-sample variability, compared to normalization using endogenous controls [60], our main results are reported here using the exogenous control ath-miR-159.

### 4.5. Statistical Analyses

Analyses utilized R software version 2.14.1. Mann–Whitney tests were used to compare plasma miRNA expression levels between cases and controls. miRNA expression profiles were analyzed independently and according to tumor histological subtype, using Elastic net and Mann–Whitney test. False Discovery Rate (FDR) was calculated using Benjamini–Hochberg (BH) correction, and results were verified using Benjamini–Yekutieli (BY), implemented in R with the padjust function to adjust for multiple comparisons; Bonferroni correction was also used as a recommended adjustment for independent covariates [61,62]. Reported FDR values are BY corrected.

Statistically significant upregulated miRNAs in cases versus controls were selected for signature identification. Three statistical approaches were used for signature identification: C-Statistics, Elastic Net, and Maximizing R Square Algorithm (MARSA). C-Statistics is useful to determine the predictive ability of individual miRNAs to identify cases. In this analysis, the FC increase was calculated as the ratio between the median plasma miRNA expression in the “cancer” group (cases, LUAD and LUSC combined) versus controls (healthy individuals). miRNAs with the highest C-statistics (≥0.9) were selected, and a weighted average was calculated with the coefficients from the univariable logistic regression as weights. When two miRNAs had the same high C-statistics values, the miRNA with the highest fold increase was chosen. These data were applied to Elastic Net, in which miRNA expression values were log_2_-transformed and then z-standardized. The function glmnet within the glmnet package was applied [63,64,65,66], and the lambda and alpha parameters were obtained through five-fold cross-validation. A modified version of MARSA was also used [67,68]. To evaluate whether plasma miRNA signatures had prognostic potential, Receiver Operating Characteristic (ROC) curves and Areas Under the Curves (AUC) were calculated. All tests were two-sided, with significance level set at *p* < 0.01. Venn diagrams were generated using jvenn viewer (http://jvenn.toulouse.inra.fr/app/example.html) [69].

### 4.6. Computational Data Analyses

Differentially expressed miRNAs were integrated with previously published datasets. This analysis used an existing number of 48.6 million miRNA-target gene interactions identified in the microRNA Data Integration Portal (miRDIP) (http://ophid.utoronto.ca/mirDIP/) [32], and miRNA-target gene interactions were validated by miRTarBase [70]. Next, we analyzed deregulated miRNAs and validated the consistency of differential expression of their targets. Comparison of our data with multiple publicly available gene expression datasets generated in tumor tissue allowed us to identify consistently deregulated genes in LUAD and LUSC. We then assembled an interaction network between deregulated miRNAs and their target genes, including transcription factors and tyrosine kinases participating in these interactions. Following this analysis, we compared the target genes of miRNAs that composed the three signatures with lung cancer deregulated genes reported in the Cancer Genome Atlas (TCGA) database studies [71]. Hierarchical clustering analysis was performed using the mean expression levels [log2 (TPM+1)] of lung miRNA target genes in LUAD and LUSC, and compared with expression in normal tissues (*n* = 383) available in the GTEx database (http://www.gtexportal.org/) [33]. We used the Scipy library in Python, by applying the cosine distance and average linkage in the clustergrammer tool, available at http://amp.pharm.mssm.edu/clustergrammer/ [72]. Similar analysis strategies have been previously reported [73,74,75]. Additionally, Enrichr (http://amp.pharm.mssm.edu/Enrichr) [76] was used as a comprehensive search tool, to determine the biological relevance of genes targeted by miRNAs, expressed in normal lung, and deregulated in lung cancer tissues.

## 5. Conclusions

The present study provides supporting evidence to the existing literature, of potential utility of circulating miRNA signatures for early detection of lung cancer. miRNAs included in the C-Statistics signature are predicted to modulate genes and pathways with known roles in lung tumorigenesis, suggesting that these miRNAs are biologically relevant in adenocarcinoma and squamous cell carcinoma of the lung.

## Figures and Tables

**Table 1:** Top 10 miRNAs with high levels in patient plasma, by statistical significance.

miRNA (Ranked by *p*-Value)	Patients (Cases)	Healthy (Controls)	FC	*p* Value of FC	FDR
**Lung adenocarcinoma (LUAD)**					
*miR-16-5p*	1852.9	381.9	4.85	2.563 × 10^−6^	0.002
*miR-451a*	14,300.9	2518.2	5.68	2.319 × 10^−4^	0.035
*miR-92a-3p*	528.4	154.4	3.42	4.203 × 10^−4^	0.035
*miR-25-3p*	408.4	87.5	4.67	1.508 × 10^−3^	0.035
miR-494-3p	4293.8	327.5	13.11	2.918 × 10^−3^	0.035
*miR-1285-5p*	55.4	5.6	9.79	2.918 × 10^−3^	0.035
miR-125b-5p	42.0	8.5	4.90	2.918 × 10^−3^	0.035
miR-448	16.0	1.3	11.73	3.587 × 10^−3^	0.035
*miR-155-5p*	112.5	15.1	7.45	3.587 × 10^−3^	0.035
miR-2682-5p	93.2	9.8	9.43	4.377 × 10^−3^	0.035
**Lung squamous cell carcinoma (LUSC)**					
*miR-16-5p*	2043.0	381.9	5.35	2.447 × 10^−4^	0.153
*miR-451a*	17,129.5	2518.2	6.80	2.676 × 10^−3^	0.153
*miR-92a-3p*	394.3	154.4	2.55	3.276 × 10^−3^	0.153
*miR-25-3p*	499.4	87.5	5.71	4.056 × 10^−3^	0.153
miR-149-5p	141.5	16.3	8.68	4.601 × 10^−3^	0.153
miR-548ah-5p	45.7	6.1	7.50	4.601 × 10^−3^	0.153
*miR-1285-5p*	36.3	5.6	6.42	4.601 × 10^−3^	0.153
*miR-155-5p*	89.4	15.1	5.92	4.601 × 10^−3^	0.153
miR-3168	12.1	2.2	5.38	4.601 × 10^−3^	0.153
miR-575	17.5	1.6	10.99	5.939 × 10^−3^	0.153

Values shown are the median levels of miRNAs in cases and controls. FC: fold change; FDR: false discovery rate; italicized miRNAs are common to both LUAD and LUSC; Underlined miRNAs are the ones incorporated in the C-Statistics signature and selected for target prediction and pathway analyses. *p*-values were obtained by Mann–Whitney test.

**Table 2:** Detected levels of the 12 miRNAs identified from the three signatures (discovery set), ranked by a combined score, which includes the averages of the ranked fold change (largest to smallest), *p* value (smallest to largest), and C-Statistics (largest to smallest). The lower the combined score, the more significant.

MiRNA	Patients (Cases)	Healthy (Controls)	FC	*p* Value of FC	C-Statistics	Combined Score
*miR-16-5p*	1886.09	381.98	4.94	1.98 × 10^−6^	0.97	2.00
*miR-451a*	14,890.94	2518.28	5.91	0.00018	0.91	2.33
*miR-1285-5p*	48.61	5.66	8.59	0.0013	0.86	3.00
*miR-92a-3p*	516.32	154.48	3.34	0.0007	0.91	3.67
*miR-155-5p*	108.73	15.12	7.19	0.0015	0.86	4.00
miR-217	3.55	1.4	2.53	0.04	0.74	6.00
miR-378e	327.84	146.72	2.23	0.19	0.65	7.00
miR-1285-3*p*	6.6	3.61	1.83	0.54	0.57	8.33
miR-106b-5*p*	40.35	36.43	1.11	0.47	0.41	9.67
miR-664a-3*p*	10.01	9.2	1.08	0.84	0.53	9.67
miR-484	26.6	25.81	1.03	0.90	0.52	11.00
miR-148b-3*p*	39.05	37.71	1.03	0.98	0.49	11.33

Values shown are Median levels of miRNAs in cases and controls. Italicized miRNAs are among the top 10 miRNAs by statistical significance in both LUAD and LUSC discovery lists; miR-25-3*p* was not identified in any signatures. Underlined miRNAs are the ones selected for target prediction and pathway analyses. *p* values were obtained by Mann–Whitney test.

**Table 3:** Validation results for the signatures and individual miRNAs (median expression levels) in a separate cohort of lung cancer patients (cases) and healthy controls.

MiRNAs	Patients (Cases)	Healthy (Control)	FC	C-Statistics	*p* Value of FC
Signatures					
C-Statistics	0.0548	0.0155		0.727	0.00019
Elastic net	5.4338	2.7423		0.644	0.013
MARSA	0.0142	0.0042		0.644	0.013
Individual miRNAs					
*miR-16-5p*	2.205	0.8973	2.0	0.676	0.0031
*miR-92a-3p*	1.9971	0.9947	2.4	0.653	0.009
*miR-451a*	1.4331	1.0229	1.8	0.629	0.024
miR-378e	5.8682	1.132	5.1	0.726	0.0002
miR-1285-3*p*	4.8923	0.6846	7.9	0.69	0.0016
*miR-1285-5p*	2.3703	1.0443	2.7	0.701	0.00086
miR-217	6.2591	1.2573	9.5	0.766	0.000013
miR-106b-5*p*	2.5163	1.134	1.7	0.651	0.0098
miR-148b-3*p*	3.8442	0.9845	3.8	0.661	0.0063
miR-484	4.2059	1.7054	2.6	0.735	0.00011
miR-664a-3*p*	8.3679	1.185	4.7	0.739	0.000084
*miR-155-5p*	1.1978	0.8908	0.8	0.508	0.45

*p* values were obtained by Mann–Whitney test; significance level ≤0,02. Italicized miRNAs are in the top 10 miRNAs by statistical significance in both LUAD and LUSC discovery lists; miR-25-3p was not identified in any signatures. Underlined miRNAs are the ones selected for target prediction and pathway analyses.

## Supplementary Materials

The following are available online at https://www.mdpi.com/2072-6694/12/8/2071/s1, Figure S1: miRNA levels in the validation set, as determined by qRT-PCR, and normalized using the endogenous control miR-93, Figure S2: The 84 genes targeted by the C-Statistics signature miRNAs (miR-16-5p, miR-92a-3p and miR-451a) are expressed in normal lung tissues (n=383, data retrieved from the GTEx database) (panel A). The bioinformatic analyses study design: target prediction for the C-Statistics signature miRNAs, assessment of gene expression in a large dataset of normal lung tissues, pathways analysis and validation of target gene expression in LUAD and LUSC tumors from the TCGA dataset is depicted in panel B, Figure S3: Study design including samples and methods used. Study samples (patients and healthy controls) were selected and included in the discovery and validation sets. The figure outlines the study outcomes in each step, Table S1: Selected literature studies on circulating miRNAs to improve detection or diagnosis of lung cancer, Table S2: Over- and under-expressed miRNAs in plasma from patients with lung adenocarcinoma (original results obtained from the Nanostring nCounter® assay), Table S3: Over- and under-expressed miRNAs in plasma from patients with lung squamous cell carcinoma (original results obtained from the Nanostring nCounter® assay), Table S4: List of commonly over-expressed miRNAs in plasma from LUAD and LUSC patients, Table S5: Genes targeted by the C-Statistics miRNAs, and expressed in normal lung tissues, Table S6: Genes targeted by C-Statistics miRNAs, and commonly deregulated in LUAD and LUSC (TCGA dataset), Table S7: Gene ontology and pathways analysis, Table S8: Demographic and histopathological data of patients, Table S9: Oligonucleotide sequences of exogenous miRNAs used as spike in controls, Table S10: TaqMan® primer sequences used for validation experiments.
