# Supplementary material for: Circulating miR-16-5p, miR-92a-3p, and miR-451a in Plasma from Lung Cancer Patients: Potential Application in Early Detection and a Regulatory Role in Tumorigenesis Pathways
Source: Cancers (Basel). 2020 Jul 27;12(8):2071. doi: 10.3390/cancers12082071 (PMC7465670; doi:10.3390/cancers12082071)
Supplement: Supplementary file 1 [file cancers-12-02071-s001.zip › Figure S3.pptx]

## Slide 1
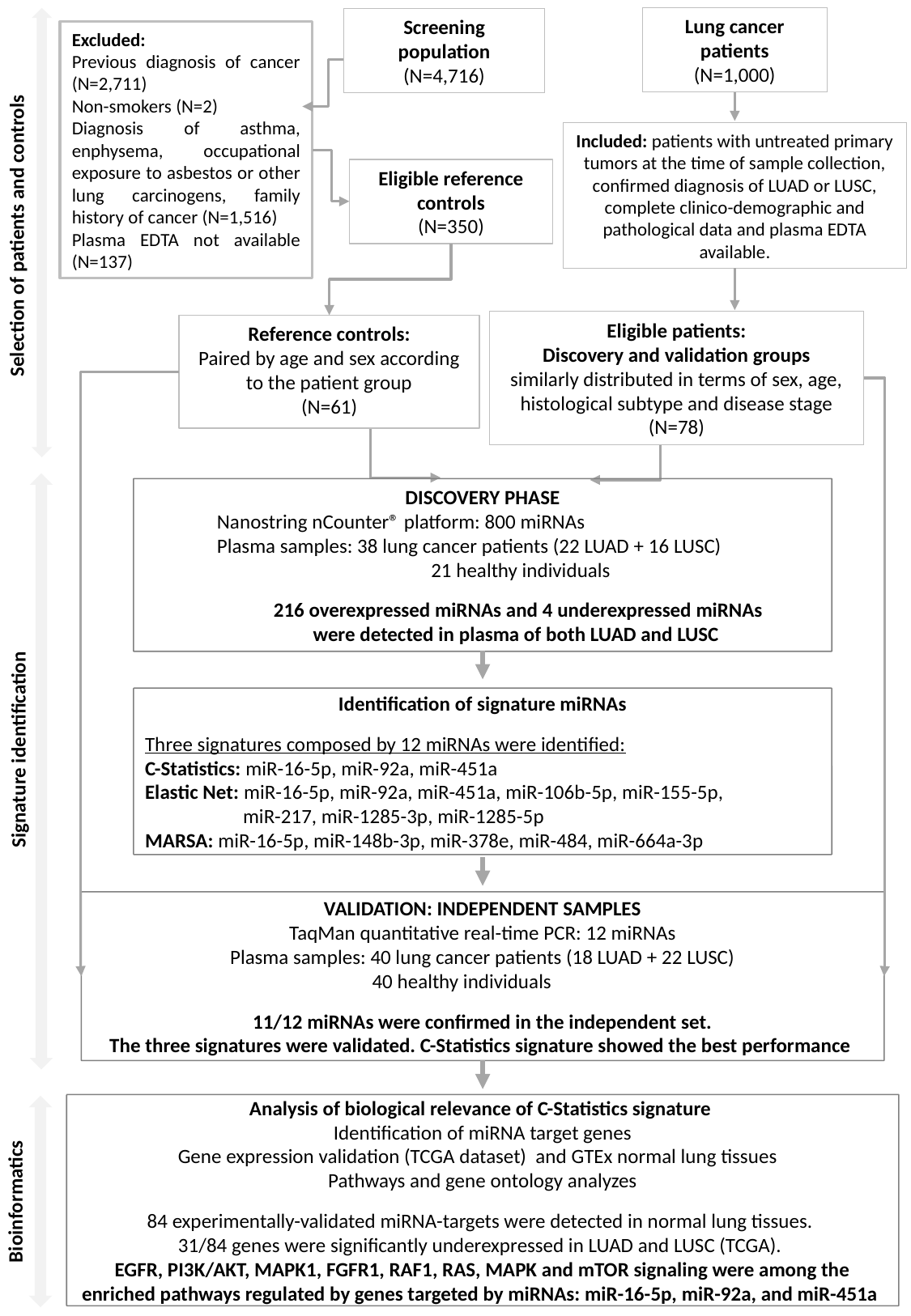

Lung cancer patients
(N=1,000)
Screening population
(N=4,716)
Excluded:
Previous diagnosis of cancer (N=2,711)
Non-smokers (N=2)
Diagnosis of asthma, enphysema, occupational exposure to asbestos or other lung carcinogens, family history of cancer (N=1,516)
Plasma EDTA not available (N=137)
Included: patients with untreated primary tumors at the time of sample collection, confirmed diagnosis of LUAD or LUSC, complete clinico-demographic and pathological data and plasma EDTA available.
Eligible reference controls
(N=350)
Selection of patients and controls
Eligible patients:
Discovery and validation groups
similarly distributed in terms of sex, age, histological subtype and disease stage
(N=78)
Reference controls:
Paired by age and sex according to the patient group
(N=61)
DISCOVERY PHASE
Nanostring nCounter® platform: 800 miRNAs
Plasma samples: 38 lung cancer patients (22 LUAD + 16 LUSC)
 21 healthy individuals
216 overexpressed miRNAs and 4 underexpressed miRNAs
were detected in plasma of both LUAD and LUSC
Identification of signature miRNAs
Three signatures composed by 12 miRNAs were identified:
C-Statistics: miR-16-5p, miR-92a, miR-451a
Elastic Net: miR-16-5p, miR-92a, miR-451a, miR-106b-5p, miR-155-5p,
 miR-217, miR-1285-3p, miR-1285-5p
MARSA: miR-16-5p, miR-148b-3p, miR-378e, miR-484, miR-664a-3p
Signature identification
VALIDATION: INDEPENDENT SAMPLES
TaqMan quantitative real-time PCR: 12 miRNAs
Plasma samples: 40 lung cancer patients (18 LUAD + 22 LUSC)
 40 healthy individuals
11/12 miRNAs were confirmed in the independent set.
The three signatures were validated. C-Statistics signature showed the best performance
Analysis of biological relevance of C-Statistics signature
Identification of miRNA target genes
Gene expression validation (TCGA dataset) and GTEx normal lung tissues
Pathways and gene ontology analyzes
84 experimentally-validated miRNA-targets were detected in normal lung tissues.
31/84 genes were significantly underexpressed in LUAD and LUSC (TCGA).
EGFR, PI3K/AKT, MAPK1, FGFR1, RAF1, RAS, MAPK and mTOR signaling were among the enriched pathways regulated by genes targeted by miRNAs: miR-16-5p, miR-92a, and miR-451a
Bioinformatics
