# Supplementary material for: Circulating miR-16-5p, miR-92a-3p, and miR-451a in Plasma from Lung Cancer Patients: Potential Application in Early Detection and a Regulatory Role in Tumorigenesis Pathways
Source: Cancers (Basel). 2020 Jul 27;12(8):2071. doi: 10.3390/cancers12082071 (PMC7465670; doi:10.3390/cancers12082071)
Supplement: Supplementary file 1 [file cancers-12-02071-s001.zip › Table S1.docx]

**Table S1.** Selected literature studies on circulating miRNAs to improve detection or diagnosis of lung cancer.

| **Study** | **Sample type and number (patients and controls)** | **Histology and Disease Stage**  **(when applicable or available)** | **Number of analyzed miRNAs/platform** | **Main results** | **Main conclusions** |
| --- | --- | --- | --- | --- | --- |
| Bianchi *et al*. 2011 [1] | Serum  Test set:64 patients  Validation set:64 patients | LUAD (n=70)  LUSC (N=25) | 365 miRNAs  TaqMan microRNA arrays (Life Technologies/Thermo Fisher) | A 34-miRNA signature was identified in the serum from patients with LUAD. Accuracy of detection of tumors in early stages: 80%. | The signature was capable of identifying disease in symptomatic and asymptomatic individuals. Also, distinguished benign from malignant lesions. The signature may be clinically relevant in lung cancer early detection programs. |
| Boeri *et al*.  2011 [2] | Plasma  Screening study including cohorts of high risk individuals: smokers: INT/IEO (n=1,035)  MILD (N=2,352) followed by LDCT scan.  Test set:INT-IEO (N=19)  Validation set: MILD (N=22) | N/A | 235 miRNAs  miRNA microarrays (Ohio State University Comprehensive Cancer Center, v. 2.0). | Cancer was diagnosed in 38 and 53 individuals (INT and MILD cohorts, respectively); miR-221, miR-660, miR-486-5p, miR-28-3p, miR-197, miR-106a, miR-451, miR-140-5p, and miR-16 have high levels associated with aggressive disease. Low levels of miR-486-5p were associated with poor prognosis. | Distinct miRNA signatures were associated with lung cancer diagnosis, prognosis, and risk of lung cancer development in high-risk individuals (chronic smokers). |
| Zheng *et al*.  2011 [3] | Plasma  74 patients  68 controls | LUAD (n=18)  LUSC (n=23)  LCC (n=7)  SCLC (n=17)  I (n=21)  II (n=12)  III (n=11)  IV (n=30) | 15 miRNAs selected from literature data on deregulated miRNAs in lung tumor tissue.  RT-QPCR. | miR-155, miR-197, and miR-182 were significantly increased in patient plasma, including stage I disease. The three miRNAs, combined, were able to differentiate plasma samples from patients *vs*. controls. miR-155 and miR-197 were further increased in plasma from patients with metastasis. | miR-155, miR-197, and miR-182 may serve as noninvasive biomarkers for early detection of lung cancer. |
| **Study** | **Sample type and number (patients and controls)** | **Histology and Disease Stage**  **(when applicable or available)** | **Number of analyzed miRNAs/platform** | **Main results** | **Main conclusions** |
| Wei *et al*.  2011 [4] | Plasma  63 patients  30 controls | LUAD (n=38)  LUSC (n=15)  LCC (n=1)  Not classified (n=9)  I-II (n=28)  IIIB-IV (n=35) | One miRNA: miR-21  RT-QPCR (SYBR Green). | miR-21 plasma levels were significantly increased in patients compared to controls, and increased in patients with tumors in advanced stages (III-IV) compared to early stage tumors (I-II). | miR-21may be useful as a plasma biomarker for early diagnosis in NSCLC. Increased miR-21 levels were associated with higher sensitivity to chemotherapy using platin-based agents. |
| Chen *et al.*  2012 [5] | Serum  400 patients  220 controls  Test and validation sets with equal number of samples. | LUAD (n=124)  LUSC (n=60)  LCC (n=16)  LUAD (n=108)  LUSC (n=75)  LCC (n=17) | 91 miRNAs were initially evaluated and then validated.  TaqManRT-QPCR. | 10 miRNAs were differentially expressed in serum from patients*vs*. controls. Risk score analysis showed that these 10 miRNAs were able to distinguish cases from controls (AUC=0.966 – test set and 0.972 – validation set). | Expression profiles for the 10 miRNAs may represent noninvasive, diagnostic biomarkers in NSCLC. |
| Aushev *et al*.  2013 [6] | Plasma  32 patients  *samples collected before and after surgery.  2 controls (patients with other tumor subtypes: one carcinoid and one sarcomatoid tumor). | LUSC (n=64 samples; 32 patients)  I-II (most patients) | 90 miRNAs including calibration controls  miRCURY LNA detection probes (Exiqon). | A specific miRNA set (miR-205, miR-19a,miR-19b, miR-30b, and miR-20a) have decreased levels in plasma from patients with LUSC, after surgery. High levels of these miRNAs were identified in tumor exosomes. | Results corroborate the notion that tumor cells secrete exosomes carrying miRNAs. The identification of global expression levels of exosome-derived miRNAscirculating in plasma may reveal novel biomarkers in cancer. |
| **Study** | **Sample type and number (patients and controls)** | **Histology and Disease Stage**  **(when applicable or available)** | **Number of analyzed miRNAs/platform** | **Main results** | **Main conclusions** |
| Sozzi *et al.*  2014 [7] | Plasma  69 patients  870 controls | NSCLC  I (n=37)  II-III (n=12)  IV (n=19) | 24 miRNAs previously identified by Boeri et al., 2011.  RT-QPCR | Validation of the 24-MSC (miRNA classifier). Results showed 87% sensitivity and 81% specificity for the 24-MSC, for detection of cancer. | The 24-MSC has diagnostic, prognostic and predictive potential. It can reduce false-positive findings of LDCT scan, improving the efficacy of early detection screening. |
| Powrózek *et al*.  2015 [8] | Plasma  90 patients  85 controls | LUAD (n=28)  LUSC (n=32)  IA, IB (n=11)  IIA, IIB (n=18)  IIIA (n=11)  IIIB (n=12)  IV (n=8)  SCLC (n=30)  IIIA (n=8)  IIIB (n=13)  IV (n=9) | Two miRNAs:  miR-944 and miR-3662  RT-QPCR | miR-944 and miR-3662 have increased levels in patient plasma.  miR-944 and miR-3662 further increased levelswere associated with more advanced disease stages, IIIB/IV, compared to stages I/II. | Lower levels of miR-944 and miR-3662 were associated with stable disease, operable tumors (LUAD and LUSC).  These miRNAs may be biomarkers for specific histological subtypes of NSCLC. |
| Sestini *et al*.  2015 [9] | Plasma  Screening cohorts: high-risk individuals (n=3,411); 84 lung cancer patients; 31 patients with plasma samples collected before and after surgery (n=100). | LUAD (n=84)  I (n=49)  II-IV (n=35) | 24 miRNA classifier (24 MSC).  Multiplex PCR, microfluidic cards (Life Technologies/Thermo Fisher). | The 24-MSC (miRNA classifier) was tested. High-risk patients (according to the 24-MSC results) showed lower 5-year survival. Patients with disease recurrence had increased risk scores associated with the time of diagnosis of recurrence. | Study data may be useful to monitor high-risk individuals for the development of lung cancer. |
|  |  |  |  |  |  |
| **Study** | **Sample type and number (patients and controls)** | **Histology and Disease Stage**  **(when applicable or available)** | **Number of analyzed miRNAs/platform** | **Main results** | **Main conclusions** |
| Wozniak *et al*. 2015 [10] | Plasma  100 patients  100 controls | LUAD (n=35)  LUSC (n=65)  IA (n=16)  IB (n=33)  IIA (n=6)  IIB (n=15)  IIIA (n=30) | 754 miRNAs  TaqMan miRNA  array (Life Technologies/  Thermo Fisher) | A panel of 24 miRNAs was able to discriminate patients and controls: let-7b, let-7c, miR-122, miR-182, miR-193a-5p, miR-200c, miR-203, miR-218, miR-155, miR-411, miR-450b-5p, miR- 485-3p, miR-519a, miR-642, miR- 517b, miR- 520f, miR-206, miR-566, miR-661, miR-340, miR-1243, miR-720, miR-543, miR-1267. | The 24 miRNAs have a high predictive power for cancer when compared to known risk factors. |
| Fan *et al*.  2016 [11] | Serum  Test set:  94 patients  58 controls  Validation set:  70 patients  54 controls | Test set:  LUAD (n=76)  LUSC (n=16)  LCC (n=2)  I (n=67)  IIA–IIIB (n=27)  Validation set:  LUAD (n=56)  LUSC (n=12)  LCC (n=2) | Seven miRNAs:  miR-15b-5p, miR-16-5p, miR-17b-5p, miR-19-3p, miR-20a-5p, miR-28-3p, miR-92-3p.  RT-QPCR used in the test set; Quantum dot fluorescence liquid bead array used in the validation set. | miRNAs were significantly increased in patient serum, except miR-15b-5p, which had decreased levels in serum from patients compared to controls.  In the validation data, combined levels for miRNAs miR-15b-5p, miR-16-5p, and miR-20a-5p showed the best performance for detection of cancer. | Detection method using quantum dots showed high sensitivity and specificity. This method is thus applicable for detection of miRNAs in body fluids. |
|  |  |  |  |  |  |
|  |  |  |  |  |  |
| **Study** | **Sample type and number (patients and controls)** | **Histology and Disease Stage**  **(when applicable or available)** | **Number of analyzed miRNAs/platform** | **Main results** | **Main conclusions** |
| Powrózek *et al*.  2016 [12] | Plasma  90 patients  85 controls | LUAD (n=30)  LUSC (n=35)  IA, IB (n=11)  IIA, IIB (n=18)  IIIA (n=11)  IIIB (n=10)  IV (n=15)  SCLC (n=25)  IIIA, IIIB (n=10)  IV (n=15) | Four miRNAs:  miR-448, miR-506,  miR-4316, and  miR-4478.  RT-QPCR | miR-448 and miR-4478 had significantly increased levels in patient plasma. | miRNAs are potential, noninvasive biomarkers in lung cancer. |
| Halvorsen *et al*.  2016 [13] | Serum  Test set  38 patients  16 controls  Validation set  107 individuals (IELCAP screening trial); 51 patients | LUAD (n=38)  IA-IB (n=24)  IIA-IIB (n=5)  IIIA (n=8)  IV (n=1) | 754 miRNAs  RT-QPCR | Expression levels of seven miRNAs classified patients and controls. Six miRNAs were validated (miR-429, miR-205, miR-200b, miR-203,  miR-125b, miR-34b) having increased levels in patient serum. | The six validated miRNAs may be useful for validation screening studies including a large number of individuals. |
| **Study** | **Sample type and number (patients and controls)** | **Histology and Disease Stage**  **(when applicable or available)** | **Number of analyzed miRNAs/platform** | **Main results** | **Main conclusions** |
| Tai *et al.*  2016 [14] | Serum  Test set  143 patients  49 controls  Validation set  110 patients  52 controls  47 patients with benign lung diseases | LUAD (n=143)  I (n=80)  II (n=21)  III (n=37)  IV (n=5)  LUAD (n=110)  I (n=65)  II (n=15)  III (n=30) | 754 miRNAs  TaqMan miRNA  arrays (Life Technologies/  Thermo Fisher) | A 20-miRNA classifier was identified. Data were validated. 90.8% of LUAD, stage I cases, were diagnosed correctly based on the levels of the 20 miRNAs. Five out of the 47 patients with benign lung diseases were false positives for cancer. | The 20-miRNA classifier may be usefulto discriminate LUAD from other lung diseases.Validation studies are needed to confirm clinical application of this classifier, including routine diagnostic use in lung cancer. |
| Arab *et al*.  2017 [15] | Plasma  Test set  34 patients (pooled samples)  20 controls  Validation set  72 patients  50 controls | Test  LUAD(n=19)  I (n=6)  II (n=4)  IIIA (n=4)  IIIB-IV (n=5)  LUSC (n=15)  I (n=3)  II (n=3)  IIIA (n=4)  IIIB-IV (n=5)  Validation  LUAD (n=41)  LUSC (n=31) | 44 miRNAs  miRCURY LNA™  microRNA PCR Panel (Exiqon) | 17 miRNAs had high levels and nine had low levels in patient plasma (test set). Of these, four miRNAs (miR-21, miR-328, miR-375 and miR-141) were selected for validation. miR-141 was the only validated miRNA. | Considering the stability of circulating miRNAs in plasma, miR-141 may be useful for inclusion in screening,validation studies, to test its potential for early detection of lung cancer. |
| **Study** | **Sample type and number (patients and controls)** | **Histology and Disease Stage**  **(when applicable or available)** | **Number of analyzed miRNAs/platform** | **Main results** | **Main conclusions** |
| Leng *et al*.  2018 [16] | Plasma  56 patients  28 controls | LUAD (n=28)  LUSC (n=28)  I (n=12)  II (n=6)  III (n=13)  IV (n=18)  Undetermined (n=7) | 11 miRNAs:  miR-205-5p, miR-145, miR-422a, miR-34a-5p, miR-93-5p, miR-223-3p, miR-210-3p, miR-628-3p, let-7d-5p, let-7g-5p, let-7i-5p  FirePlex detection assay | 10/11 miRNAs were significantly increased in patient plasma (miR-205-5p, miR-422a, miR-34a-5p, miR-93-5p, miR-223-3p, miR-210-3p, miR-628-3p, let-7d-5p, let-7g-5p, let-7i-5p). A predictive model was developed for miR-205-5p and miR-210-3p, and showed predictive power for disease detection regardless of histological subtype or disease stage. | miR-205-5p and miR-210-3p may be biomarkers for early detection of lung cancer. Furthermore, combined levels for miRNA subsets may be helpful in tumor histological classification. |
| Su *et al*.  2018 [17] | Sputum and PBMC  Test set  68 patients  66 controls  Validation set  49 patients  50 controls | Test  LUAD (n=37)  LUSC (n=31)  I - II (n=22)  III (n=20)  IV (n=26)  Validation  LUAD (n=26)  LUSC (n=23)  I (n=15)  II (n=15)  III-IV (n=19) | Five miRNAs:  miR-21, miR-31, miR-210 (sputum) and miR-19b-3p, miR-29b-3p (PBMC)  TaqMan RT-QPCR | Combined levels of miR-31 and miR-210 in sputum, and miR-19b-3p in PBMC have higher specificity and sensitivity for detection of cancer, regardless of histological subtype. | Combined levels of circulating miRNAs may represent a panel for improved early detection of lung cancer. |
|  |  |  |  |  |  |
|  |  |  |  |  |  |
| **Study** | **Sample type and number (patients and controls)** | **Histology and Disease Stage**  **(when applicable or available)** | **Number of analyzed miRNAs/platform** | **Main results** | **Main conclusions** |
| Sun *et al*.  2018 [18] | Plasma and tumor tissue  28 patients  28 controls | LUAD (n=28)  IA (n=12)  IB (n=3)  IIA (n=2)  IIB (n=5)  IIIA (n=4)  IIIB (n=2) | Two miRNAs:  miR-21 and miR-339-5p  miRNA microarrays and RT-QPCR | miR-21 levels were increased and miR-339-5p had significantly lower levels in plasma and solid tumor tissue from patients. | Plasma levels of miR-339-5p or miR-21 may serve as diagnostic biomarkers. Combined plasma levels of these miRNAs were efficient to detect lung adenocarcinoma. |
|  |  |  |  |  |  |
| Zou *et al*.  2019 [19] | Serum and tumor tissue  50 patients  30 controls | NSCLC | Three miRNAs:  miR-182, miR-200b, miR-205  RT-QPCR | Increased levels of miR-182 and miR-205 and decreased levels of miR-200b were detected in patient serum. | The combined levels of miR-182, miR-200b and miR-205 may be a useful biomarker for early diagnosis in lung cancer. |
| Yang *et al*.  2019 [20] | Serum  63 patients | Test  LUAD (n=30)  LUSC (n=33)  I (n=46)  II (n=17)  Validation  LUAD (n=36)  LUSC (n=29)  I (n=29)  II (n=16)  III (n=20) | Eight miRNAs:  miR-146b, miR-205, miR-29c, miR-31,  miR-30b, miR-337,  miR-411, and miR-708  RT-QPCR | A 4-miRNAs set (miR-146b, miR-205, miR-29c and miR-30b) was shown to have diagnostic power for NSCLC patients in the training and validation sets. | The 4-miRNA panel is suggested as a useful non-invasive serum biomarker for early diagnosis of NSCLC. |
| **Study** | **Sample type and number (patients and controls)** | **Histology and Disease Stage**  **(when applicable or available)** | **Number of analyzed miRNAs/platform** | **Main results** | **Main conclusions** |
| Hetta *et al*.  2019 [21] | Plasma  45 patients  40 controls | NSCLC | Two miRNAs:  miR-21, miR-23a | miR-21 and miR-23a were significantly over-expressed in plasma samples from patients compared to controls. | miR-21 and miR-23a may be useful targets for molecular therapies. The results require validation in larger, prospective patient cohorts with consistent methods. |

NSCLC: non-small cell lung cancer. SCLC: small cell lung cancer. LUAD: lung adenocarcinoma. LUSC: lung squamous cell carcinoma. LCC: large cell carcinoma. RT-QPCR: reverse transcription, quantitative real-time PCR. LDCT: low dose computer tomography. PBMC: peripheral blood mononuclear cells.INT/IO: *Instituto Nazionale Tumori of Milan*; MILD: Multicentric Italian Lung Detection trial; IELCAP: International Early Lung Cancer Action Program.Note that, for some studies, the number of samples in the discovery and validation sets are grouped together. N/A: not applicable.

References

1. Bianchi, F.; Nicassio, F.; Marzi, M.; Belloni, E.; Dall’Olio, V.; Bernard, L.; Pelosi, G.; Maisonneuve, P.; Veronesi, G.; Di Fiore, P.P. A serum circulating miRNA diagnostic test to identify asymptomatic high-risk individuals with early stage lung cancer. *EMBO Mol. Med.* **2011**, *3*, 495–503.

2. Boeri, M.; Verri, C.; Conte, D.; Roz, L.; Modena, P.; Facchinetti, F.; Calabrò, E.; Croce, C.M.; Pastorino, U.; Sozzi, G. MicroRNA signatures in tissues and plasma predict development and prognosis of computed tomography detected lung cancer. *Proc. Natl. Acad. Sci. U. S. A.* **2011**, *108*, 3713–3718.

3. Zheng, D.; Haddadin, S.; Wang, Y.; Gu, L.Q.; Perry, M.C.; Freter, C.E.; Wang, M.X. Plasma micrornas as novel biomarkers for early detection of lung cancer. *Int. J. Clin. Exp. Pathol.* **2011**, *4*, 575–586.

4. Wei, J.; Gao, W.; Zhu, C.J.; Liu, Y.Q.; Mei, Z.; Cheng, T.; Shu, Y.Q. Identification of plasma microRNA-21 as a biomarker for early detection and chemosensitivity of non-small cell lung cancer. *Chin. J. Cancer* **2011**, *30*, 407–414.

5. Chen, X.; Hu, Z.; Wang, W.; Ba, Y.; Ma, L.; Zhang, C.; Wang, C.; Ren, Z.; Zhao, Y.; Wu, S.; et al. Identification of ten serum microRNAs from a genome-wide serum microRNA expression profile as novel noninvasive biomarkers for nonsmall cell lung cancer diagnosis. *Int. J. Cancer* **2012**, *130*, 1620–1628.

6. Aushev, V.N.; Zborovskaya, I.B.; Laktionov, K.K.; Girard, N.; Cros, M.-P.; Herceg, Z.; Krutovskikh, V. Comparisons of microRNA Patterns in Plasma before and after Tumor Removal Reveal New Biomarkers of Lung Squamous Cell Carcinoma. *PLoS One* **2013**, *8*, e78649.

7. Sozzi, G.; Boeri, M.; Rossi, M.; Verri, C.; Suatoni, P.; Bravi, F.; Roz, L.; Conte, D.; Grassi, M.; Sverzellati, N.; et al. Clinical utility of a plasma-based miRNA signature classifier within computed tomography lung cancer screening: A correlative MILD trial study. *J. Clin. Oncol.* **2014**, *32*, 768–773.

8. Powrózek, T.; Krawczyk, P.; Kowalski, D.M.; Winiarczyk, K.; Olszyna-Serementa, M.; Milanowski, J. Plasma circulating microRNA-944 and microRNA-3662 as potential histologic type-specific early lung cancer biomarkers. *Transl. Res.* **2015**, *166*, 315–323.

9. Sestini, S.; Boeri, M.; Marchiano, A.; Pelosi, G.; Galeone, C.; Verri, C.; Suatoni, P.; Sverzellati, N.; Vecchia, C. La; Sozzi, G.; et al. Circulating microRNA signature as liquid-biopsy to monitor lung cancer in low-dose computed tomography screening. *Oncotarget* **2015**, *6*, 32868–32877.

10. Wozniak, M.B.; Scelo, G.; Muller, D.C.; Mukeria, A.; Zaridze, D.; Brennan, P. Circulating MicroRNAs as Non-Invasive Biomarkers for Early Detection of Non-Small-Cell Lung Cancer. *PLoS One* **2015**, *10*, e0125026.

11. Fan, L.; Qi, H.; Teng, J.; Su, B.; Chen, H.; Wang, C.; Xia, Q. Identification of serum miRNAs by nano-quantum dots microarray as diagnostic biomarkers for early detection of non-small cell lung cancer. *Tumor Biol.* **2016**, *37*, 7777–7784.

12. Powrózek, T.; Krawczyk, P.; Kowalski, D.M.; Kuźnar-Kamińska, B.; Winiarczyk, K.; Olszyna-Serementa, M.; Batura-Gabryel, H.; Milanowski, J. Application of plasma circulating microRNA-448, 506, 4316, and 4478 analysis for non-invasive diagnosis of lung cancer. *Tumour Biol.* **2016**, *37*, 2049–55.

13. Halvorsen, A.R.; Bjaanæs, M.; LeBlanc, M.; Holm, A.M.; Bolstad, N.; Rubio, L.; Peñalver, J.C.; Cervera, J.; Mojarrieta, J.C.; López-Guerrero, J.A.; et al. A unique set of 6 circulating microRNAs for early detection of non-small cell lung cancer. *Oncotarget* **2016**, *7*, 37250–37259.

14. Tai, M.C.; Yanagisawa, K.; Nakatochi, M.; Hotta, N.; Hosono, Y.; Kawaguchi, K.; Naito, M.; Taniguchi, H.; Wakai, K.; Yokoi, K.; et al. Blood-borne miRNA profile-based diagnostic classifier for lung adenocarcinoma. *Sci. Rep.* **2016**, *6*, 31389.

15. Arab, A.; Karimipoor, M.; Irani, S.; Kiani, A.; Zeinali, S.; Tafsiri, E.; Sheikhy, K. Potential circulating miRNA signature for early detection of NSCLC. *Cancer Genet.* **2017**, *216*–*217*, 150–158.

16. Leng, Q.; Wang, Y.; Jiang, F. A Direct Plasma miRNA Assay for Early Detection and Histological Classification of Lung Cancer. *Transl. Oncol.* **2018**, *11*, 883–889.

17. Su, J.; Leng, Q.; Lin, Y.; Ma, J.; Jiang, F.; Lee, C.-J.; Fang, H.; Jiang, F. Integrating Circulating Immunological and Sputum Biomarkers for the Early Detection of Lung Cancer. *Biomark. Cancer* **2018**, *10*, 1179299X18759297.

18. Sun, Y.; Mei, H.; Xu, C.; Tang, H.; Wei, W. Circulating microRNA-339-5p and -21 in plasma as an early detection predictors of lung adenocarcinoma. *Pathol. Res. Pract.* **2018**, *214*, 119–125.

19. Zou, J.G.; Ma, L.F.; Li, X.; Xu, F.L.; Fei, X.Z.; Liu, Q.; Bai, Q.L.; Dong, Y.L. Circulating microRNA array (miR-182, 200b and 205) for the early diagnosis and poor prognosis predictor of non-small cell lung cancer. *Eur. Rev. Med. Pharmacol. Sci.* **2019**, *23*, 1108–1115.

20. Yang, X.; Zhang, Q.; Zhang, M.; Su, W.; Wang, Z.; Li, Y.; Zhang, J.; Beer, D.G.; Yang, S.; Chen, G. Serum microRNA signature is capable of early diagnosis for non-small cell lung cancer. *Int. J. Biol. Sci.* **2019**, *15*, 1712–1722.

21. Hetta, H.F.; Zahran, A.M.; Shafik, E.A.; El-Mahdy, R.I.; Mohamed, N.A.; Nabil, E.E.; Esmaeel, H.M.; Alkady, O.A.; Elkady, A.; Mohareb, D.A.; et al. Circulating miRNA-21 and miRNA-23a Expression Signature as Potential Biomarkers for Early Detection of Non-Small-Cell Lung Cancer. *MicroRNA* **2019**, *8*, 206–215.
