# Supplementary material for: Circulating miR-16-5p, miR-92a-3p, and miR-451a in Plasma from Lung Cancer Patients: Potential Application in Early Detection and a Regulatory Role in Tumorigenesis Pathways
Source: Cancers (Basel). 2020 Jul 27;12(8):2071. doi: 10.3390/cancers12082071 (PMC7465670; doi:10.3390/cancers12082071)
Supplement: Supplementary file 1 [file cancers-12-02071-s001.zip › Table S2.docx]

**Table S2.** Over- and under-expressed miRNAs in plasma from patients with lung adenocarcinoma. These are the original results obtained from the Nanostring nCounter® assay.

| **miRNA** | **Patient plasma (LUAD)** | **Healthy controls plasma** | ***Fold change*** | ***p*** |
| --- | --- | --- | --- | --- |
| ***Over-expressed miRNAs*** |  |  |  |  |
| miR-16-5p | 1852.900 | 381.980 | 4.85 | 2.563E-06 |
| miR-451a | 14300.985 | 2518.280 | 5.68 | 2.319E-04 |
| miR-92a-3p | 528.470 | 154.480 | 3.42 | 4.203E-04 |
| miR-25-3p | 408.430 | 87.530 | 4.67 | 1.508E-03 |
| miR-494-3p | 4293.885 | 327.560 | 13.11 | 2.918E-03 |
| miR-1285-5p | 55.405 | 5.660 | 9.79 | 2.918E-03 |
| miR-125b-5p | 42.090 | 8.590 | 4.90 | 2.918E-03 |
| miR-448 | 16.070 | 1.370 | 11.73 | 3.587E-03 |
| miR-155-5p | 112.580 | 15.120 | 7.45 | 3.587E-03 |
| miR-2682-5p | 93.215 | 9.880 | 9.43 | 4.377E-03 |
| miR-548j-3p | 55.690 | 6.120 | 9.10 | 4.377E-03 |
| miR-378i | 79.110 | 9.960 | 7.94 | 4.377E-03 |
| miR-574-5p | 183.240 | 24.890 | 7.36 | 4.377E-03 |
| miR-873-3p | 23.005 | 3.180 | 7.23 | 4.377E-03 |
| miR-197-5p | 10.120 | 1.420 | 7.13 | 4.377E-03 |
| miR-323b-3p | 13.455 | 2.130 | 6.32 | 4.377E-03 |
| miR-216a-5p | 7.910 | 1.500 | 5.27 | 4.377E-03 |
| miR-876-3p | 3.675 | 1.000 | 3.68 | 4.620E-03 |
| miR-548al | 9.625 | 1.400 | 6.88 | 4.671E-03 |
| miR-3065-5p | 292.120 | 35.810 | 8.16 | 5.314E-03 |
| miR-149-5p | 129.065 | 16.300 | 7.92 | 5.314E-03 |
| miR-625-5p | 54.965 | 8.240 | 6.67 | 5.314E-03 |
| miR-612 | 101.640 | 15.540 | 6.54 | 5.314E-03 |
| miR-1323 | 14.175 | 2.550 | 5.56 | 5.314E-03 |
| miR-150-5p | 168.685 | 30.870 | 5.46 | 5.314E-03 |
| miR-1283 | 2166.930 | 396.930 | 5.46 | 5.314E-03 |
| miR-1180-3p | 18.780 | 3.500 | 5.37 | 5.314E-03 |
| miR-133a-5p | 8.090 | 1.000 | 8.09 | 5.356E-03 |
| miR-515-5p | 5.340 | 1.000 | 5.34 | 5.356E-03 |
| miR-501-3p | 2.885 | 1.000 | 2.89 | 5.362E-03 |
| miR-564 | 3.850 | 1.000 | 3.85 | 5.419E-03 |
| miR-1226-3p | 3.580 | 1.000 | 3.58 | 5.419E-03 |
| miR-566 | 5.465 | 1.170 | 4.67 | 5.454E-03 |
| miR-296-5p | 17.710 | 1.990 | 8.90 | 5.471E-03 |
| miR-1908-3p | 5.290 | 1.000 | 5.29 | 6.269E-03 |
| miR-6724-5p | 3.440 | 1.000 | 3.44 | 6.269E-03 |
| miR-3150b-3p | 3.405 | 1.000 | 3.41 | 6.269E-03 |
| miR-505-3p | 3.110 | 1.000 | 3.11 | 6.269E-03 |
| miR-506-5p | 3.950 | 1.000 | 3.95 | 6.327E-03 |
| miR-153-3p | 5.720 | 1.000 | 5.72 | 6.334E-03 |
| miR-1307-5p | 7.925 | 1.330 | 5.96 | 6.373E-03 |
| miR-3144-3p | 112.820 | 8.950 | 12.61 | 6.392E-03 |
| miR-628-5p | 11.455 | 1.350 | 8.49 | 6.392E-03 |
| miR-378h | 52.395 | 5.500 | 9.53 | 6.411E-03 |
| miR-337-3p | 105.200 | 11.230 | 9.37 | 6.411E-03 |
| miR-3613-3p | 19.310 | 2.200 | 8.78 | 6.411E-03 |
| miR-1290 | 95.375 | 11.890 | 8.02 | 6.411E-03 |
| miR-610 | 67.035 | 8.700 | 7.71 | 6.411E-03 |
| miR-1268a | 23.895 | 4.530 | 5.27 | 6.411E-03 |
| miR-30e-5p | 193.710 | 46.230 | 4.19 | 6.411E-03 |
| miR-548y | 32.025 | 3.230 | 9.91 | 6.904E-03 |
| miR-3168 | 20.790 | 2.260 | 9.20 | 6.904E-03 |
| miR-572 | 4.315 | 1.000 | 4.32 | 7.313E-03 |
| miR-181d-3p | 3.440 | 1.000 | 3.44 | 7.313E-03 |
| miR-548k | 11.025 | 1.000 | 11.03 | 7.385E-03 |
| miR-190a-3p | 4.620 | 1.000 | 4.62 | 7.385E-03 |
| miR-92b-3p | 3.685 | 1.000 | 3.69 | 7.385E-03 |
| miR-4443 | 17.885 | 1.500 | 11.92 | 7.451E-03 |
| miR-301b-3p | 14.610 | 1.790 | 8.16 | 7.451E-03 |
| miR-196a-5p | 68.200 | 7.580 | 9.00 | 7.695E-03 |
| miR-526a+miR-518c-5p+miR-518d-5p | 23.995 | 2.700 | 8.89 | 7.695E-03 |
| miR-1255b-5p | 17.675 | 1.990 | 8.88 | 7.695E-03 |
| miR-520h | 91.590 | 11.480 | 7.98 | 7.695E-03 |
| miR-548ah-5p | 40.860 | 6.100 | 6.70 | 7.695E-03 |
| miR-411-5p | 32.735 | 4.990 | 6.56 | 7.695E-03 |
| miR-3161 | 49.730 | 8.670 | 5.74 | 7.695E-03 |
| miR-195-5p | 9.670 | 2.080 | 4.65 | 7.695E-03 |
| miR-877-5p | 32.210 | 10.510 | 3.06 | 7.695E-03 |
| miR-1266-5p | 8.110 | 1.000 | 8.11 | 7.776E-03 |
| miR-582-5p | 3.440 | 1.000 | 3.44 | 7.776E-03 |
| miR-615-3p | 3.440 | 1.000 | 3.44 | 7.776E-03 |
| miR-604 | 3.395 | 1.000 | 3.40 | 7.776E-03 |
| miR-514a-3p | 3.155 | 1.000 | 3.16 | 7.776E-03 |
| miR-3690 | 4.550 | 1.000 | 4.55 | 8.509E-03 |
| miR-203a-5p | 3.780 | 1.000 | 3.78 | 8.509E-03 |
| miR-99a-5p | 3.685 | 1.000 | 3.69 | 8.509E-03 |
| miR-1275 | 3.440 | 1.000 | 3.44 | 8.509E-03 |
| miR-302a-3p | 2.950 | 1.000 | 2.95 | 8.591E-03 |
| miR-517c-3p+miR-519a-3p | 5.930 | 1.170 | 5.07 | 8.640E-03 |
| miR-1973 | 4.275 | 1.290 | 3.31 | 8.640E-03 |
| miR-511-5p | 10.670 | 1.420 | 7.51 | 8.665E-03 |
| miR-219a-1-3p | 3.685 | 1.000 | 3.69 | 9.033E-03 |
| miR-515-3p | 10.415 | 1.000 | 10.42 | 9.042E-03 |
| miR-591 | 6.715 | 1.000 | 6.72 | 9.042E-03 |
| miR-3196 | 5.185 | 1.000 | 5.19 | 9.042E-03 |
| miR-520d-3p | 5.070 | 1.000 | 5.07 | 9.042E-03 |
| miR-2053 | 4.275 | 1.000 | 4.28 | 9.042E-03 |
| miR-4647 | 3.850 | 1.000 | 3.85 | 9.042E-03 |
| miR-4425 | 3.770 | 1.000 | 3.77 | 9.042E-03 |
| miR-10b-5p | 3.580 | 1.000 | 3.58 | 9.042E-03 |
| miR-596 | 3.545 | 1.000 | 3.55 | 9.042E-03 |
| miR-3180 | 3.405 | 1.000 | 3.41 | 9.042E-03 |
| miR-519e-3p | 3.405 | 1.000 | 3.41 | 9.042E-03 |
| miR-1270 | 3.110 | 1.000 | 3.11 | 9.042E-03 |
| miR-3934-5p | 3.110 | 1.000 | 3.11 | 9.042E-03 |
| miR-4741 | 2.795 | 1.000 | 2.80 | 9.042E-03 |
| miR-599 | 2.795 | 1.000 | 2.80 | 9.042E-03 |
| miR-6511a-5p | 2.795 | 1.000 | 2.80 | 9.042E-03 |
| miR-935 | 2.535 | 1.000 | 2.54 | 9.042E-03 |
| miR-128-1-5p | 56.530 | 7.180 | 7.87 | 9.185E-03 |
| miR-1257 | 69.940 | 9.200 | 7.60 | 9.185E-03 |
| miR-4536-5p | 87.675 | 11.770 | 7.45 | 9.185E-03 |
| miR-1206 | 34.125 | 4.820 | 7.08 | 9.185E-03 |
| miR-514b-5p | 78.630 | 12.240 | 6.42 | 9.185E-03 |
| miR-1322 | 42.155 | 7.770 | 5.43 | 9.185E-03 |
| miR-4286 | 53.690 | 13.360 | 4.02 | 9.185E-03 |
| miR-654-5p | 3.590 | 1.080 | 3.32 | 9.257E-03 |
| miR-889-3p | 3.325 | 1.080 | 3.08 | 9.301E-03 |
| miR-455-5p | 4.835 | 1.000 | 4.84 | 9.878E-03 |
| miR-1272 | 4.540 | 1.000 | 4.54 | 9.878E-03 |
| miR-181b-2-3p | 3.505 | 1.000 | 3.51 | 9.878E-03 |
| miR-5001-3p | 3.395 | 1.000 | 3.40 | 9.878E-03 |
| miR-517a-3p | 3.280 | 1.000 | 3.28 | 9.878E-03 |
| miR-654-3p | 3.280 | 1.000 | 3.28 | 9.878E-03 |
| miR-382-3p | 3.200 | 1.000 | 3.20 | 9.878E-03 |
| miR-375 | 5.250 | 1.000 | 5.25 | 9.969E-03 |
| miR-651-5p | 4.890 | 1.000 | 4.89 | 9.969E-03 |
| miR-922 | 4.255 | 1.000 | 4.26 | 9.969E-03 |
| miR-1287-3p | 3.685 | 1.000 | 3.69 | 9.969E-03 |
| miR-1288-3p | 2.795 | 1.000 | 2.80 | 9.969E-03 |
| miR-513a-3p | 7.435 | 1.420 | 5.24 | 1.002E-02 |
| miR-212-3p | 16.345 | 1.850 | 8.84 | 1.005E-02 |
| miR-874-5p | 23.200 | 2.970 | 7.81 | 1.005E-02 |
| miR-188-5p | 10.145 | 1.710 | 5.93 | 1.005E-02 |
| miR-766-5p | 2.850 | 1.000 | 2.85 | 1.048E-02 |
| miR-214-3p | 2.585 | 1.000 | 2.59 | 1.048E-02 |
| miR-367-3p | 5.845 | 1.000 | 5.85 | 1.049E-02 |
| miR-208b-3p | 4.190 | 1.000 | 4.19 | 1.049E-02 |
| miR-708-5p | 3.850 | 1.000 | 3.85 | 1.049E-02 |
| miR-1269a | 3.615 | 1.000 | 3.62 | 1.049E-02 |
| miR-224-5p | 3.545 | 1.000 | 3.55 | 1.049E-02 |
| miR-767-3p | 3.395 | 1.000 | 3.40 | 1.049E-02 |
| miR-1234-3p | 3.200 | 1.000 | 3.20 | 1.049E-02 |
| miR-299-5p | 3.110 | 1.000 | 3.11 | 1.049E-02 |
| miR-301b-5p | 3.110 | 1.000 | 3.11 | 1.049E-02 |
| miR-3131 | 3.110 | 1.000 | 3.11 | 1.049E-02 |
| miR-520e | 3.110 | 1.000 | 3.11 | 1.049E-02 |
| miR-1233-3p | 3.075 | 1.000 | 3.08 | 1.049E-02 |
| miR-380-3p | 2.950 | 1.000 | 2.95 | 1.049E-02 |
| miR-25-5p | 2.795 | 1.000 | 2.80 | 1.049E-02 |
| miR-4707-3p | 2.795 | 1.000 | 2.80 | 1.049E-02 |
| miR-524-3p | 2.795 | 1.000 | 2.80 | 1.049E-02 |
| miR-532-3p | 2.795 | 1.000 | 2.80 | 1.049E-02 |
| miR-675-5p | 2.795 | 1.000 | 2.80 | 1.049E-02 |
| miR-758-5p | 2.795 | 1.000 | 2.80 | 1.049E-02 |
| miR-1248 | 2.535 | 1.000 | 2.54 | 1.049E-02 |
| miR-202-3p | 2.535 | 1.000 | 2.54 | 1.049E-02 |
| miR-508-5p | 2.535 | 1.000 | 2.54 | 1.049E-02 |
| miR-553 | 4.550 | 1.080 | 4.21 | 1.078E-02 |
| miR-1185-2-3p | 30.550 | 1.920 | 15.91 | 1.091E-02 |
| miR-644a | 111.610 | 12.510 | 8.92 | 1.091E-02 |
| miR-548n | 90.345 | 11.270 | 8.02 | 1.091E-02 |
| miR-548v | 17.115 | 2.260 | 7.57 | 1.091E-02 |
| miR-613 | 43.340 | 6.740 | 6.43 | 1.091E-02 |
| miR-585-3p | 57.625 | 9.200 | 6.26 | 1.091E-02 |
| miR-489-3p | 18.710 | 3.110 | 6.02 | 1.091E-02 |
| miR-363-5p | 23.235 | 4.850 | 4.79 | 1.091E-02 |
| miR-652-5p | 3.395 | 1.000 | 3.40 | 1.143E-02 |
| miR-1293 | 4.220 | 1.000 | 4.22 | 1.144E-02 |
| miR-642a-5p | 3.840 | 1.000 | 3.84 | 1.144E-02 |
| miR-548b-3p | 3.395 | 1.000 | 3.40 | 1.144E-02 |
| miR-891a-5p | 6.940 | 1.000 | 6.94 | 1.154E-02 |
| miR-545-3p | 3.405 | 1.000 | 3.41 | 1.154E-02 |
| miR-942-5p | 3.765 | 1.420 | 2.65 | 1.160E-02 |
| miR-330-3p | 2.535 | 1.000 | 2.54 | 1.213E-02 |
| miR-615-5p | 3.685 | 1.000 | 3.69 | 1.214E-02 |
| miR-187-3p | 3.440 | 1.000 | 3.44 | 1.214E-02 |
| miR-576-5p | 3.110 | 1.000 | 3.11 | 1.214E-02 |
| miR-552-3p | 3.075 | 1.000 | 3.08 | 1.214E-02 |
| miR-1271-3p | 2.795 | 1.000 | 2.80 | 1.214E-02 |
| miR-211-3p | 2.795 | 1.000 | 2.80 | 1.214E-02 |
| miR-346 | 2.795 | 1.000 | 2.80 | 1.214E-02 |
| miR-595 | 2.795 | 1.000 | 2.80 | 1.214E-02 |
| miR-664b-3p | 2.795 | 1.000 | 2.80 | 1.214E-02 |
| miR-371b-5p | 2.600 | 1.000 | 2.60 | 1.214E-02 |
| miR-3192-5p | 2.585 | 1.000 | 2.59 | 1.214E-02 |
| miR-3180-3p | 2.535 | 1.000 | 2.54 | 1.214E-02 |
| miR-487b-5p | 2.535 | 1.000 | 2.54 | 1.214E-02 |
| miR-517b-3p | 2.535 | 1.000 | 2.54 | 1.214E-02 |
| miR-4451 | 6.835 | 1.330 | 5.14 | 1.241E-02 |
| miR-551a | 6.045 | 1.350 | 4.48 | 1.241E-02 |
| miR-512-5p | 4.880 | 1.000 | 4.88 | 1.281E-02 |
| miR-510-3p | 4.495 | 1.000 | 4.50 | 1.281E-02 |
| miR-1228-3p | 3.850 | 1.000 | 3.85 | 1.281E-02 |
| miR-3918 | 3.615 | 1.000 | 3.62 | 1.281E-02 |
| miR-137 | 3.440 | 1.000 | 3.44 | 1.281E-02 |
| miR-30a-3p | 3.405 | 1.000 | 3.41 | 1.281E-02 |
| miR-34c-5p | 3.090 | 1.000 | 3.09 | 1.281E-02 |
| miR-590-3p | 2.795 | 1.000 | 2.80 | 1.281E-02 |
| miR-769-3p | 2.795 | 1.000 | 2.80 | 1.281E-02 |
| miR-362-3p | 2.535 | 1.000 | 2.54 | 1.281E-02 |
| miR-422a | 695.855 | 92.300 | 7.54 | 1.290E-02 |
| miR-1915-3p | 24.905 | 3.640 | 6.84 | 1.290E-02 |
| miR-577 | 16.295 | 2.550 | 6.39 | 1.290E-02 |
| miR-3136-5p | 49.910 | 9.830 | 5.08 | 1.290E-02 |
| miR-486-3p | 30.270 | 6.160 | 4.91 | 1.290E-02 |
| miR-378f | 9.405 | 2.260 | 4.16 | 1.290E-02 |
| miR-100-5p | 6.865 | 1.000 | 6.87 | 1.320E-02 |
| miR-381-5p | 5.070 | 1.000 | 5.07 | 1.322E-02 |
| miR-939-5p | 4.810 | 1.000 | 4.81 | 1.322E-02 |
| miR-499b-3p | 4.785 | 1.000 | 4.79 | 1.322E-02 |
| miR-339-3p | 3.395 | 1.000 | 3.40 | 1.322E-02 |
| miR-147a | 2.795 | 1.000 | 2.80 | 1.322E-02 |
| miR-3074-3p | 2.795 | 1.000 | 2.80 | 1.322E-02 |
| miR-548o-3p+miR-548ah-3p+miR-548av-3p | 2.795 | 1.000 | 2.80 | 1.322E-02 |
| miR-571 | 2.795 | 1.000 | 2.80 | 1.322E-02 |
| miR-576-3p | 2.535 | 1.000 | 2.54 | 1.322E-02 |
| miR-499a-5p | 17.215 | 1.420 | 12.12 | 1.343E-02 |
| miR-1252-5p | 2.820 | 1.000 | 2.82 | 1.418E-02 |
| miR-504-3p | 3.405 | 1.170 | 2.91 | 1.431E-02 |
| miR-1972 | 11.460 | 1.830 | 6.26 | 1.442E-02 |
| miR-587 | 51.055 | 10.370 | 4.92 | 1.442E-02 |
| miR-518d-3p | 3.395 | 1.000 | 3.40 | 1.477E-02 |
| miR-607 | 5.160 | 1.000 | 5.16 | 1.478E-02 |
| miR-450b-3p | 4.605 | 1.000 | 4.61 | 1.478E-02 |
| miR-616-3p | 4.190 | 1.000 | 4.19 | 1.478E-02 |
| miR-1304-5p | 3.950 | 1.000 | 3.95 | 1.478E-02 |
| miR-4458 | 3.825 | 1.000 | 3.83 | 1.478E-02 |
| miR-196a-3p | 3.545 | 1.000 | 3.55 | 1.478E-02 |
| miR-4536-3p | 3.440 | 1.000 | 3.44 | 1.478E-02 |
| miR-200b-3p | 3.405 | 1.000 | 3.41 | 1.478E-02 |
| miR-378b | 3.395 | 1.000 | 3.40 | 1.478E-02 |
| miR-507 | 3.395 | 1.000 | 3.40 | 1.478E-02 |
| miR-556-3p | 3.395 | 1.000 | 3.40 | 1.478E-02 |
| miR-582-3p | 3.395 | 1.000 | 3.40 | 1.478E-02 |
| miR-1304-3p | 3.110 | 1.000 | 3.11 | 1.478E-02 |
| miR-499b-5p | 3.110 | 1.000 | 3.11 | 1.478E-02 |
| miR-520b | 3.110 | 1.000 | 3.11 | 1.478E-02 |
| miR-542-3p | 3.110 | 1.000 | 3.11 | 1.478E-02 |
| miR-614 | 3.110 | 1.000 | 3.11 | 1.478E-02 |
| miR-1302 | 2.950 | 1.000 | 2.95 | 1.478E-02 |
| miR-103a-3p | 2.795 | 1.000 | 2.80 | 1.478E-02 |
| miR-1224-5p | 2.795 | 1.000 | 2.80 | 1.478E-02 |
| miR-133b | 2.795 | 1.000 | 2.80 | 1.478E-02 |
| miR-2113 | 2.795 | 1.000 | 2.80 | 1.478E-02 |
| miR-298 | 2.795 | 1.000 | 2.80 | 1.478E-02 |
| miR-338-5p | 2.795 | 1.000 | 2.80 | 1.478E-02 |
| miR-429 | 2.795 | 1.000 | 2.80 | 1.478E-02 |
| miR-518e-3p | 2.795 | 1.000 | 2.80 | 1.478E-02 |
| miR-605-5p | 2.795 | 1.000 | 2.80 | 1.478E-02 |
| miR-648 | 2.795 | 1.000 | 2.80 | 1.478E-02 |
| miR-936 | 2.795 | 1.000 | 2.80 | 1.478E-02 |
| miR-548c-5p+miR-548o-5p+miR-548am-5p | 2.790 | 1.000 | 2.79 | 1.478E-02 |
| miR-514b-3p | 2.725 | 1.000 | 2.73 | 1.478E-02 |
| miR-205-5p | 2.585 | 1.000 | 2.59 | 1.478E-02 |
| miR-128-2-5p | 2.535 | 1.000 | 2.54 | 1.478E-02 |
| miR-134-3p | 2.535 | 1.000 | 2.54 | 1.478E-02 |
| miR-300 | 2.535 | 1.000 | 2.54 | 1.478E-02 |
| miR-512-3p | 2.535 | 1.000 | 2.54 | 1.478E-02 |
| miR-580-3p | 2.535 | 1.000 | 2.54 | 1.478E-02 |
| miR-637 | 2.535 | 1.000 | 2.54 | 1.478E-02 |
| miR-548ad-3p | 20.260 | 1.920 | 10.55 | 1.518E-02 |
| miR-302a-5p | 26.025 | 3.540 | 7.35 | 1.518E-02 |
| miR-2117 | 18.010 | 2.610 | 6.90 | 1.518E-02 |
| miR-548g-3p | 75.330 | 12.050 | 6.25 | 1.518E-02 |
| miR-556-5p | 130.220 | 22.200 | 5.87 | 1.518E-02 |
| miR-320b | 3.590 | 1.000 | 3.59 | 1.522E-02 |
| miR-1278 | 3.110 | 1.000 | 3.11 | 1.522E-02 |
| miR-568 | 7.665 | 1.000 | 7.67 | 1.523E-02 |
| miR-514a-5p | 4.480 | 1.000 | 4.48 | 1.523E-02 |
| miR-181a-2-3p | 3.325 | 1.000 | 3.33 | 1.523E-02 |
| miR-3164 | 3.200 | 1.000 | 3.20 | 1.523E-02 |
| miR-199b-5p | 3.135 | 1.000 | 3.14 | 1.523E-02 |
| miR-509-5p | 4.730 | 1.080 | 4.38 | 1.535E-02 |
| miR-2110 | 4.620 | 1.000 | 4.62 | 1.536E-02 |
| miR-663a | 3.850 | 1.000 | 3.85 | 1.536E-02 |
| miR-551b-3p | 5.500 | 1.500 | 3.67 | 1.542E-02 |
| miR-192-5p | 14.050 | 1.580 | 8.89 | 1.543E-02 |
| miR-626 | 10.140 | 1.330 | 7.62 | 1.543E-02 |
| miR-215-5p | 8.880 | 1.600 | 5.55 | 1.543E-02 |
| miR-10a-5p | 4.755 | 1.420 | 3.35 | 1.543E-02 |
| miR-1305 | 17.885 | 2.610 | 6.85 | 1.547E-02 |
| miR-563 | 12.325 | 1.420 | 8.68 | 1.647E-02 |
| miR-151b | 3.405 | 1.170 | 2.91 | 1.647E-02 |
| miR-620 | 3.045 | 1.000 | 3.05 | 1.700E-02 |
| miR-455-3p | 2.795 | 1.000 | 2.80 | 1.700E-02 |
| miR-383-5p | 2.600 | 1.000 | 2.60 | 1.700E-02 |
| miR-1200 | 2.535 | 1.000 | 2.54 | 1.700E-02 |
| miR-490-5p | 2.535 | 1.000 | 2.54 | 1.700E-02 |
| miR-608 | 7.380 | 1.000 | 7.38 | 1.702E-02 |
| miR-1185-5p | 4.190 | 1.000 | 4.19 | 1.702E-02 |
| miR-449c-5p | 4.140 | 1.000 | 4.14 | 1.702E-02 |
| miR-548ak | 3.985 | 1.000 | 3.99 | 1.702E-02 |
| miR-624-3p | 3.845 | 1.000 | 3.85 | 1.702E-02 |
| miR-345-5p | 3.405 | 1.000 | 3.41 | 1.702E-02 |
| miR-548d-5p | 3.405 | 1.000 | 3.41 | 1.702E-02 |
| miR-940 | 3.405 | 1.000 | 3.41 | 1.702E-02 |
| miR-323a-5p | 3.395 | 1.000 | 3.40 | 1.702E-02 |
| miR-328-5p | 3.395 | 1.000 | 3.40 | 1.702E-02 |
| miR-365b-5p | 3.395 | 1.000 | 3.40 | 1.702E-02 |
| miR-518c-3p | 3.395 | 1.000 | 3.40 | 1.702E-02 |
| miR-523-3p | 3.395 | 1.000 | 3.40 | 1.702E-02 |
| miR-548e-3p | 3.395 | 1.000 | 3.40 | 1.702E-02 |
| miR-875-3p | 3.395 | 1.000 | 3.40 | 1.702E-02 |
| miR-550a-5p | 3.145 | 1.000 | 3.15 | 1.702E-02 |
| miR-1224-3p | 3.110 | 1.000 | 3.11 | 1.702E-02 |
| miR-127-5p | 3.110 | 1.000 | 3.11 | 1.702E-02 |
| miR-412-3p | 3.110 | 1.000 | 3.11 | 1.702E-02 |
| miR-492 | 3.110 | 1.000 | 3.11 | 1.702E-02 |
| miR-542-5p | 3.110 | 1.000 | 3.11 | 1.702E-02 |
| miR-548h-5p | 3.110 | 1.000 | 3.11 | 1.702E-02 |
| miR-603 | 3.110 | 1.000 | 3.11 | 1.702E-02 |
| miR-655-3p | 3.110 | 1.000 | 3.11 | 1.702E-02 |
| miR-892a | 3.110 | 1.000 | 3.11 | 1.702E-02 |
| miR-4792 | 3.045 | 1.000 | 3.05 | 1.702E-02 |
| miR-1202 | 2.795 | 1.000 | 2.80 | 1.702E-02 |
| miR-124-3p | 2.795 | 1.000 | 2.80 | 1.702E-02 |
| miR-1245b-3p | 2.795 | 1.000 | 2.80 | 1.702E-02 |
| miR-1247-5p | 2.795 | 1.000 | 2.80 | 1.702E-02 |
| miR-1250-5p | 2.795 | 1.000 | 2.80 | 1.702E-02 |
| miR-1254 | 2.795 | 1.000 | 2.80 | 1.702E-02 |
| miR-135b-5p | 2.795 | 1.000 | 2.80 | 1.702E-02 |
| miR-152-5p | 2.795 | 1.000 | 2.80 | 1.702E-02 |
| miR-193b-3p | 2.795 | 1.000 | 2.80 | 1.702E-02 |
| miR-208a-3p | 2.795 | 1.000 | 2.80 | 1.702E-02 |
| miR-31-5p | 2.795 | 1.000 | 2.80 | 1.702E-02 |
| miR-3179 | 2.795 | 1.000 | 2.80 | 1.702E-02 |
| miR-320c | 2.795 | 1.000 | 2.80 | 1.702E-02 |
| miR-329-3p | 2.795 | 1.000 | 2.80 | 1.702E-02 |
| miR-370-5p | 2.795 | 1.000 | 2.80 | 1.702E-02 |
| miR-371a-5p | 2.795 | 1.000 | 2.80 | 1.702E-02 |
| miR-3916 | 2.795 | 1.000 | 2.80 | 1.702E-02 |
| miR-483-5p | 2.795 | 1.000 | 2.80 | 1.702E-02 |
| miR-5010-5p | 2.795 | 1.000 | 2.80 | 1.702E-02 |
| miR-502-3p | 2.795 | 1.000 | 2.80 | 1.702E-02 |
| miR-513a-5p | 2.795 | 1.000 | 2.80 | 1.702E-02 |
| miR-513c-5p | 2.795 | 1.000 | 2.80 | 1.702E-02 |
| miR-541-3p | 2.795 | 1.000 | 2.80 | 1.702E-02 |
| miR-554 | 2.795 | 1.000 | 2.80 | 1.702E-02 |
| miR-573 | 2.795 | 1.000 | 2.80 | 1.702E-02 |
| miR-942-3p | 2.795 | 1.000 | 2.80 | 1.702E-02 |
| miR-3147 | 2.600 | 1.000 | 2.60 | 1.702E-02 |
| miR-449a | 2.585 | 1.000 | 2.59 | 1.702E-02 |
| miR-539-3p | 2.585 | 1.000 | 2.59 | 1.702E-02 |
| miR-3151-5p | 2.575 | 1.000 | 2.58 | 1.702E-02 |
| miR-561-3p | 2.575 | 1.000 | 2.58 | 1.702E-02 |
| miR-101-3p | 2.535 | 1.000 | 2.54 | 1.702E-02 |
| miR-1178-3p | 2.535 | 1.000 | 2.54 | 1.702E-02 |
| miR-1204 | 2.535 | 1.000 | 2.54 | 1.702E-02 |
| miR-1249-5p | 2.535 | 1.000 | 2.54 | 1.702E-02 |
| miR-1269b | 2.535 | 1.000 | 2.54 | 1.702E-02 |
| miR-129-5p | 2.535 | 1.000 | 2.54 | 1.702E-02 |
| miR-134-5p+miR-6728-5p | 2.535 | 1.000 | 2.54 | 1.702E-02 |
| miR-146b-3p | 2.535 | 1.000 | 2.54 | 1.702E-02 |
| miR-182-5p | 2.535 | 1.000 | 2.54 | 1.702E-02 |
| miR-208b-5p | 2.535 | 1.000 | 2.54 | 1.702E-02 |
| miR-210-5p | 2.535 | 1.000 | 2.54 | 1.702E-02 |
| miR-221-5p | 2.535 | 1.000 | 2.54 | 1.702E-02 |
| miR-2278 | 2.535 | 1.000 | 2.54 | 1.702E-02 |
| miR-297 | 2.535 | 1.000 | 2.54 | 1.702E-02 |
| miR-3140-3p | 2.535 | 1.000 | 2.54 | 1.702E-02 |
| miR-3140-5p | 2.535 | 1.000 | 2.54 | 1.702E-02 |
| miR-323b-5p | 2.535 | 1.000 | 2.54 | 1.702E-02 |
| miR-330-5p | 2.535 | 1.000 | 2.54 | 1.702E-02 |
| miR-369-5p | 2.535 | 1.000 | 2.54 | 1.702E-02 |
| miR-378c | 2.535 | 1.000 | 2.54 | 1.702E-02 |
| miR-431-5p | 2.535 | 1.000 | 2.54 | 1.702E-02 |
| miR-4448 | 2.535 | 1.000 | 2.54 | 1.702E-02 |
| miR-449b-5p | 2.535 | 1.000 | 2.54 | 1.702E-02 |
| miR-485-5p | 2.535 | 1.000 | 2.54 | 1.702E-02 |
| miR-510-5p | 2.535 | 1.000 | 2.54 | 1.702E-02 |
| miR-520a-3p | 2.535 | 1.000 | 2.54 | 1.702E-02 |
| miR-589-5p | 2.535 | 1.000 | 2.54 | 1.702E-02 |
| miR-627-5p | 2.535 | 1.000 | 2.54 | 1.702E-02 |
| miR-651-3p | 2.535 | 1.000 | 2.54 | 1.702E-02 |
| miR-664b-5p | 2.535 | 1.000 | 2.54 | 1.702E-02 |
| miR-760 | 2.535 | 1.000 | 2.54 | 1.702E-02 |
| miR-765 | 2.535 | 1.000 | 2.54 | 1.702E-02 |
| miR-887-5p | 2.535 | 1.000 | 2.54 | 1.702E-02 |
| miR-892b | 2.535 | 1.000 | 2.54 | 1.702E-02 |
| miR-924 | 2.535 | 1.000 | 2.54 | 1.702E-02 |
| miR-92a-1-5p | 2.535 | 1.000 | 2.54 | 1.702E-02 |
| miR-934 | 2.535 | 1.000 | 2.54 | 1.702E-02 |
| miR-329-5p | 4.385 | 1.000 | 4.39 | 1.731E-02 |
| miR-1203 | 3.405 | 1.000 | 3.41 | 1.731E-02 |
| miR-4461 | 4.965 | 1.000 | 4.97 | 1.751E-02 |
| miR-579-5p | 3.325 | 1.000 | 3.33 | 1.751E-02 |
| miR-1197 | 3.155 | 1.000 | 3.16 | 1.751E-02 |
| miR-210-3p | 3.395 | 1.330 | 2.55 | 1.774E-02 |
| miR-4531 | 142.860 | 17.250 | 8.28 | 1.778E-02 |
| miR-888-5p | 41.510 | 5.910 | 7.02 | 1.778E-02 |
| miR-520d-5p+miR-527+miR-518a-5p | 217.240 | 32.730 | 6.64 | 1.778E-02 |
| miR-4516 | 37.950 | 13.470 | 2.82 | 1.778E-02 |
| miR-1295a | 14.965 | 2.130 | 7.03 | 1.778E-02 |
| miR-190a-5p | 3.825 | 1.000 | 3.83 | 1.855E-02 |
| miR-6503-5p | 3.405 | 1.000 | 3.41 | 1.855E-02 |
| miR-34c-3p | 4.550 | 1.090 | 4.17 | 1.891E-02 |
| miR-642a-3p | 3.440 | 1.330 | 2.59 | 1.891E-02 |
| miR-575 | 13.210 | 1.600 | 8.26 | 1.904E-02 |
| miR-499a-3p | 5.250 | 1.000 | 5.25 | 1.986E-02 |
| miR-190b | 4.115 | 1.000 | 4.12 | 1.986E-02 |
| miR-617 | 4.070 | 1.000 | 4.07 | 1.986E-02 |
| miR-326 | 3.395 | 1.000 | 3.40 | 1.986E-02 |
| miR-188-3p | 3.110 | 1.000 | 3.11 | 1.986E-02 |
| miR-376a-2-5p | 3.110 | 1.000 | 3.11 | 1.986E-02 |
| miR-3185 | 2.795 | 1.000 | 2.80 | 1.986E-02 |
| miR-296-3p | 2.585 | 1.000 | 2.59 | 1.986E-02 |
| miR-619-3p | 2.535 | 1.000 | 2.54 | 1.986E-02 |
| miR-373-3p | 3.280 | 1.000 | 3.28 | 2.008E-02 |
| miR-521 | 3.075 | 1.000 | 3.08 | 2.008E-02 |
| miR-3202 | 13.750 | 1.000 | 13.75 | 2.009E-02 |
| miR-183-5p | 7.905 | 1.000 | 7.91 | 2.009E-02 |
| miR-520g-3p | 3.840 | 1.000 | 3.84 | 2.009E-02 |
| miR-638 | 3.780 | 1.000 | 3.78 | 2.009E-02 |
| miR-198 | 3.405 | 1.000 | 3.41 | 2.009E-02 |
| miR-181a-3p | 3.395 | 1.000 | 3.40 | 2.009E-02 |
| miR-548e-5p | 3.395 | 1.000 | 3.40 | 2.009E-02 |
| miR-495-5p | 12.245 | 1.420 | 8.62 | 2.034E-02 |
| miR-1286 | 10.325 | 1.420 | 7.27 | 2.034E-02 |
| miR-1205 | 4.840 | 1.420 | 3.41 | 2.034E-02 |
| miR-194-5p | 5.125 | 1.420 | 3.61 | 2.039E-02 |
| miR-7-5p | 18.240 | 2.700 | 6.76 | 2.074E-02 |
| miR-630 | 41.135 | 16.590 | 2.48 | 2.074E-02 |
| miR-649 | 4.540 | 1.000 | 4.54 | 2.126E-02 |
| miR-96-5p | 5.105 | 1.400 | 3.65 | 2.166E-02 |
| miR-219b-3p | 7.520 | 1.420 | 5.30 | 2.179E-02 |
| miR-1297 | 16.070 | 2.340 | 6.87 | 2.181E-02 |
| miR-6721-5p | 7.980 | 1.580 | 5.05 | 2.181E-02 |
| miR-3615 | 4.440 | 1.000 | 4.44 | 2.274E-02 |
| miR-3144-5p | 4.315 | 1.000 | 4.32 | 2.274E-02 |
| miR-184 | 3.985 | 1.000 | 3.99 | 2.274E-02 |
| miR-548m | 3.845 | 1.000 | 3.85 | 2.274E-02 |
| miR-33b-5p | 3.675 | 1.000 | 3.68 | 2.274E-02 |
| miR-1-5p | 3.405 | 1.000 | 3.41 | 2.274E-02 |
| miR-509-3-5p | 3.110 | 1.000 | 3.11 | 2.274E-02 |
| miR-639 | 3.110 | 1.000 | 3.11 | 2.274E-02 |
| miR-1273c | 2.795 | 1.000 | 2.80 | 2.274E-02 |
| miR-1296-5p | 2.795 | 1.000 | 2.80 | 2.274E-02 |
| miR-3190-3p | 2.795 | 1.000 | 2.80 | 2.274E-02 |
| miR-376c-5p | 2.795 | 1.000 | 2.80 | 2.274E-02 |
| miR-4431 | 2.795 | 1.000 | 2.80 | 2.274E-02 |
| miR-135a-5p | 2.535 | 1.000 | 2.54 | 2.274E-02 |
| miR-1910-3p | 2.535 | 1.000 | 2.54 | 2.274E-02 |
| miR-494-5p | 2.535 | 1.000 | 2.54 | 2.274E-02 |
| miR-503-3p | 2.535 | 1.000 | 2.54 | 2.274E-02 |
| miR-606 | 2.535 | 1.000 | 2.54 | 2.274E-02 |
| miR-1279 | 4.615 | 1.000 | 4.62 | 2.298E-02 |
| miR-33a-5p | 3.440 | 1.000 | 3.44 | 2.299E-02 |
| miR-1262 | 2.950 | 1.000 | 2.95 | 2.299E-02 |
| miR-3614-3p | 3.075 | 1.000 | 3.08 | 2.315E-02 |
| miR-219a-5p | 6.465 | 1.420 | 4.55 | 2.327E-02 |
| miR-640 | 16.380 | 1.420 | 11.54 | 2.330E-02 |
| miR-324-3p | 4.820 | 1.420 | 3.39 | 2.332E-02 |
| miR-2116-5p | 178.250 | 22.670 | 7.86 | 2.409E-02 |
| miR-1287-5p | 10.400 | 2.550 | 4.08 | 2.409E-02 |
| miR-496 | 42.350 | 10.860 | 3.90 | 2.409E-02 |
| miR-342-3p | 39.950 | 14.130 | 2.83 | 2.409E-02 |
| miR-1185-1-3p | 3.110 | 1.000 | 3.11 | 2.429E-02 |
| miR-548ar-5p | 3.110 | 1.000 | 3.11 | 2.431E-02 |
| miR-203a-3p | 14.505 | 1.420 | 10.21 | 2.475E-02 |
| miR-450a-2-3p | 5.250 | 1.400 | 3.75 | 2.475E-02 |
| miR-141-3p | 5.290 | 1.420 | 3.73 | 2.475E-02 |
| miR-1236-3p | 3.850 | 1.420 | 2.71 | 2.475E-02 |
| miR-506-3p | 3.440 | 1.290 | 2.67 | 2.475E-02 |
| miR-561-5p | 3.405 | 1.420 | 2.40 | 2.475E-02 |
| miR-4787-3p | 6.315 | 1.420 | 4.45 | 2.484E-02 |
| miR-1281 | 3.110 | 1.290 | 2.41 | 2.484E-02 |
| miR-1255a | 22.920 | 2.110 | 10.86 | 2.491E-02 |
| miR-891b | 7.595 | 1.600 | 4.75 | 2.491E-02 |
| miR-3127-5p | 3.395 | 1.000 | 3.40 | 2.557E-02 |
| miR-1245a | 3.685 | 1.000 | 3.69 | 2.598E-02 |
| miR-3180-5p | 4.190 | 1.000 | 4.19 | 2.625E-02 |
| miR-345-3p | 4.550 | 1.290 | 3.53 | 2.625E-02 |
| miR-516b-5p | 3.405 | 1.000 | 3.41 | 2.625E-02 |
| miR-519c-3p | 3.045 | 1.000 | 3.05 | 2.625E-02 |
| miR-650 | 2.795 | 1.080 | 2.59 | 2.625E-02 |
| miR-6503-3p | 2.575 | 1.000 | 2.58 | 2.625E-02 |
| miR-4284 | 3.610 | 1.000 | 3.61 | 2.644E-02 |
| miR-3614-5p | 5.160 | 1.420 | 3.63 | 2.655E-02 |
| miR-365a-3p+miR-365b-3p | 13.530 | 1.500 | 9.02 | 2.661E-02 |
| miR-219a-2-3p | 18.025 | 1.830 | 9.85 | 2.787E-02 |
| miR-873-5p | 14.250 | 2.550 | 5.59 | 2.787E-02 |
| miR-656-3p | 33.695 | 7.150 | 4.71 | 2.787E-02 |
| miR-122-5p | 371.920 | 92.280 | 4.03 | 2.787E-02 |
| miR-495-3p | 31.650 | 8.380 | 3.78 | 2.787E-02 |
| miR-206 | 8.035 | 2.260 | 3.56 | 2.787E-02 |
| miR-758-3p+miR-411-3p | 3.685 | 1.420 | 2.60 | 2.822E-02 |
| miR-937-3p | 3.110 | 1.290 | 2.41 | 2.822E-02 |
| miR-1268b | 4.410 | 1.000 | 4.41 | 2.918E-02 |
| miR-204-5p | 3.440 | 1.000 | 3.44 | 2.960E-02 |
| miR-1249-3p | 3.145 | 1.000 | 3.15 | 2.960E-02 |
| miR-369-3p | 2.950 | 1.000 | 2.95 | 2.960E-02 |
| miR-3613-5p | 2.795 | 1.000 | 2.80 | 2.988E-02 |
| miR-4524a-5p | 5.250 | 1.170 | 4.49 | 2.990E-02 |
| miR-4707-5p | 3.845 | 1.000 | 3.85 | 2.990E-02 |
| miR-433-5p | 3.395 | 1.000 | 3.40 | 2.990E-02 |
| miR-1258 | 3.155 | 1.000 | 3.16 | 2.990E-02 |
| miR-362-5p | 3.110 | 1.000 | 3.11 | 2.990E-02 |
| miR-6720-3p | 2.795 | 1.000 | 2.80 | 2.990E-02 |
| miR-452-5p | 3.440 | 1.330 | 2.59 | 2.990E-02 |
| miR-631 | 2.535 | 1.000 | 2.54 | 3.011E-02 |
| miR-525-5p | 4.325 | 1.420 | 3.05 | 3.021E-02 |
| miR-548l | 5.170 | 1.170 | 4.42 | 3.023E-02 |
| miR-1298-5p | 4.880 | 1.600 | 3.05 | 3.023E-02 |
| miR-217 | 3.405 | 1.400 | 2.43 | 3.023E-02 |
| miR-138-5p | 29.495 | 4.920 | 5.99 | 3.029E-02 |
| miR-885-3p | 2.795 | 1.000 | 2.80 | 3.157E-02 |
| miR-1183 | 11.730 | 1.420 | 8.26 | 3.210E-02 |
| miR-1827 | 6.975 | 1.420 | 4.91 | 3.210E-02 |
| miR-147b | 6.690 | 1.830 | 3.66 | 3.210E-02 |
| miR-516a-3p+miR-516b-3p | 3.685 | 1.420 | 2.60 | 3.210E-02 |
| miR-450b-5p | 2.795 | 1.090 | 2.56 | 3.210E-02 |
| miR-146b-5p | 3.405 | 1.400 | 2.43 | 3.210E-02 |
| miR-301a-5p | 16.255 | 2.410 | 6.74 | 3.211E-02 |
| miR-548a-5p | 35.630 | 5.290 | 6.74 | 3.211E-02 |
| miR-216b-5p | 37.150 | 7.680 | 4.84 | 3.211E-02 |
| miR-518f-3p | 5.255 | 1.420 | 3.70 | 3.229E-02 |
| miR-1909-3p | 2.795 | 1.000 | 2.80 | 3.319E-02 |
| miR-3130-3p | 2.795 | 1.000 | 2.80 | 3.319E-02 |
| miR-3065-3p | 2.795 | 1.000 | 2.80 | 3.365E-02 |
| miR-661 | 2.950 | 1.000 | 2.95 | 3.398E-02 |
| miR-488-3p | 3.640 | 1.350 | 2.70 | 3.398E-02 |
| miR-641 | 3.110 | 1.370 | 2.27 | 3.398E-02 |
| miR-4485-3p | 5.740 | 1.420 | 4.04 | 3.421E-02 |
| miR-384 | 2.950 | 1.000 | 2.95 | 3.421E-02 |
| miR-4421 | 11.940 | 1.420 | 8.41 | 3.434E-02 |
| miR-3158-3p | 3.955 | 1.290 | 3.07 | 3.434E-02 |
| miR-133a-3p | 3.985 | 1.420 | 2.81 | 3.440E-02 |
| miR-1289 | 3.325 | 1.090 | 3.05 | 3.643E-02 |
| miR-34b-3p | 3.110 | 1.080 | 2.88 | 3.643E-02 |
| miR-562 | 5.250 | 1.580 | 3.32 | 3.663E-02 |
| miR-497-5p | 4.470 | 1.420 | 3.15 | 3.663E-02 |
| miR-592 | 5.250 | 1.830 | 2.87 | 3.663E-02 |
| miR-424-5p | 7.385 | 2.870 | 2.57 | 3.687E-02 |
| miR-1271-5p | 2.795 | 1.000 | 2.80 | 3.767E-02 |
| miR-526b-5p | 2.795 | 1.000 | 2.80 | 3.767E-02 |
| miR-9-5p | 2.795 | 1.000 | 2.80 | 3.767E-02 |
| miR-513c-3p | 2.585 | 1.000 | 2.59 | 3.767E-02 |
| miR-125a-3p | 4.190 | 1.000 | 4.19 | 3.817E-02 |
| miR-887-3p | 2.535 | 1.000 | 2.54 | 3.817E-02 |
| miR-105-5p | 3.110 | 1.000 | 3.11 | 3.853E-02 |
| miR-643 | 3.405 | 1.330 | 2.56 | 3.853E-02 |
| miR-491-5p | 5.500 | 1.000 | 5.50 | 3.877E-02 |
| miR-302e | 8.595 | 1.790 | 4.80 | 3.891E-02 |
| miR-1299 | 4.550 | 1.420 | 3.20 | 3.891E-02 |
| miR-802 | 5.070 | 1.920 | 2.64 | 3.891E-02 |
| miR-378e | 542.930 | 146.720 | 3.70 | 3.899E-02 |
| miR-140-3p | 7.250 | 2.240 | 3.24 | 3.899E-02 |
| miR-378g | 3.685 | 1.420 | 2.60 | 3.899E-02 |
| miR-584-5p | 4.315 | 1.420 | 3.04 | 4.137E-02 |
| miR-519b-5p+miR-519c-5p+miR-523-5p+miR-518e-5p+miR-522-5p+miR-519a-5p | 15.460 | 1.790 | 8.64 | 4.147E-02 |
| miR-548ai+miR-570-5p | 17.670 | 2.410 | 7.33 | 4.217E-02 |
| miR-548q | 68.835 | 15.390 | 4.47 | 4.217E-02 |
| miR-4455 | 25.905 | 8.440 | 3.07 | 4.217E-02 |
| miR-144-3p | 39.535 | 14.900 | 2.65 | 4.217E-02 |
| miR-660-3p | 4.495 | 1.000 | 4.50 | 4.265E-02 |
| miR-761 | 2.795 | 1.000 | 2.80 | 4.265E-02 |
| miR-142-5p | 2.535 | 1.000 | 2.54 | 4.265E-02 |
| miR-23c | 2.535 | 1.000 | 2.54 | 4.265E-02 |
| miR-500a-5p+miR-501-5p | 3.780 | 1.000 | 3.78 | 4.319E-02 |
| miR-139-5p | 2.950 | 1.000 | 2.95 | 4.319E-02 |
| miR-325 | 3.405 | 1.000 | 3.41 | 4.356E-02 |
| miR-450a-1-3p | 6.540 | 1.090 | 6.00 | 4.358E-02 |
| miR-374a-3p | 3.405 | 1.330 | 2.56 | 4.358E-02 |
| miR-3605-3p | 2.535 | 1.000 | 2.54 | 4.358E-02 |
| miR-520a-5p | 15.670 | 1.790 | 8.75 | 4.397E-02 |
| miR-508-3p | 11.715 | 1.600 | 7.32 | 4.400E-02 |
| miR-4755-5p | 5.395 | 1.400 | 3.85 | 4.400E-02 |
| miR-660-5p | 3.440 | 1.080 | 3.19 | 4.400E-02 |
| miR-320d | 3.685 | 1.370 | 2.69 | 4.400E-02 |
| miR-1301-3p | 3.440 | 1.420 | 2.42 | 4.400E-02 |
| miR-522-3p | 5.540 | 2.080 | 2.66 | 4.408E-02 |
| miR-519b-3p | 3.145 | 1.000 | 3.15 | 4.591E-02 |
| miR-4488 | 9.165 | 2.110 | 4.34 | 4.683E-02 |
| miR-885-5p | 9.860 | 2.260 | 4.36 | 4.807E-02 |
| miR-601 | 15.160 | 3.600 | 4.21 | 4.807E-02 |
| miR-26b-5p | 51.205 | 22.760 | 2.25 | 4.807E-02 |
| miR-1303 | 3.440 | 1.000 | 3.44 | 4.876E-02 |
| miR-548ar-3p | 3.110 | 1.000 | 3.11 | 4.876E-02 |
| miR-372-3p | 2.535 | 1.000 | 2.54 | 4.876E-02 |
| miR-5001-5p | 3.325 | 1.370 | 2.43 | 4.916E-02 |
| miR-767-5p | 3.685 | 1.000 | 3.69 | 4.918E-02 |
| miR-1291 | 3.440 | 1.000 | 3.44 | 4.918E-02 |
| miR-378d | 3.440 | 1.290 | 2.67 | 4.918E-02 |
| miR-516a-5p | 3.395 | 1.330 | 2.55 | 4.918E-02 |
| miR-182-3p | 7.710 | 1.420 | 5.43 | 4.963E-02 |
| miR-193a-5p+miR-193b-5p | 6.535 | 1.420 | 4.60 | 4.963E-02 |
| miR-5010-3p | 7.680 | 2.080 | 3.69 | 4.963E-02 |
| miR-1307-3p | 5.650 | 1.420 | 3.98 | 4.972E-02 |
| miR-152-3p | 3.685 | 1.420 | 2.60 | 4.972E-02 |
| ***Under-expressed miRNAs*** |  |  |  |  |
| miR-374a-5p | 5.940 | 34.290 | 0.17 | 1.893E-03 |
| miR-376c-3p | 4.550 | 19.770 | 0.23 | 2.359E-03 |
| miR-126-3p | 165.625 | 404.140 | 0.41 | 2.918E-03 |
| hsa-let-7g-5p | 33.330 | 100.960 | 0.33 | 3.587E-03 |
| hsa-let-7i-5p | 31.840 | 76.520 | 0.42 | 3.587E-03 |
| miR-181a-5p | 7.400 | 24.040 | 0.31 | 4.377E-03 |
| miR-146a-5p | 44.265 | 129.960 | 0.34 | 4.377E-03 |
| miR-361-5p | 4.440 | 53.510 | 0.08 | 5.314E-03 |
| miR-223-3p | 242.845 | 736.420 | 0.33 | 5.314E-03 |
| miR-15b-5p | 16.380 | 122.270 | 0.13 | 6.392E-03 |
| miR-340-5p | 5.240 | 31.730 | 0.17 | 6.411E-03 |
| miR-199a-3p+miR-199b-3p | 41.585 | 188.470 | 0.22 | 7.695E-03 |
| miR-23a-3p | 95.585 | 313.600 | 0.30 | 7.695E-03 |
| hsa-let-7a-5p | 59.045 | 279.820 | 0.21 | 9.185E-03 |
| miR-221-3p | 32.640 | 130.800 | 0.25 | 1.091E-02 |
| miR-142-3p | 46.105 | 129.960 | 0.35 | 1.290E-02 |
| miR-335-5p | 3.440 | 16.320 | 0.21 | 1.518E-02 |
| miR-382-5p | 4.550 | 17.740 | 0.26 | 1.518E-02 |
| miR-125a-5p | 5.465 | 25.600 | 0.21 | 1.778E-02 |
| miR-191-5p | 46.830 | 153.850 | 0.30 | 1.778E-02 |
| miR-409-3p | 4.550 | 23.140 | 0.20 | 2.409E-02 |
| miR-130a-3p | 55.730 | 229.470 | 0.24 | 2.409E-02 |
| miR-199a-5p | 10.345 | 36.850 | 0.28 | 2.409E-02 |
| miR-27b-3p | 8.725 | 26.290 | 0.33 | 3.211E-02 |
| miR-145-5p | 6.325 | 14.780 | 0.43 | 3.211E-02 |
| miR-24-3p | 36.720 | 74.720 | 0.49 | 3.687E-02 |

*Note that a few miRNAs are combined in the same probe in the Nanostring assay.
