# Supplementary material for: Circulating miR-16-5p, miR-92a-3p, and miR-451a in Plasma from Lung Cancer Patients: Potential Application in Early Detection and a Regulatory Role in Tumorigenesis Pathways
Source: Cancers (Basel). 2020 Jul 27;12(8):2071. doi: 10.3390/cancers12082071 (PMC7465670; doi:10.3390/cancers12082071)
Supplement: Supplementary file 1 [file cancers-12-02071-s001.zip › Table S3.docx]

**Table S3.** Over- and under-expressed miRNAs in plasma from patients with lung squamous cell carcinoma. These are the original results obtained from the Nanostring nCounter® assay.

| **miRNA** | **Patient plasma (LUSC)** | **Healthy controls plasma** | ***Fold change*** | ***p*** |
| --- | --- | --- | --- | --- |
| ***Over-expressed miRNAs*** |  |  |  |  |
| miR-16-5p | 2043.005 | 381.980 | 5.35 | 2.447E-04 |
| miR-451a | 17129.515 | 2518.280 | 6.80 | 2.676E-03 |
| miR-92a-3p | 394.325 | 154.480 | 2.55 | 3.276E-03 |
| miR-25-3p | 499.440 | 87.530 | 5.71 | 4.056E-03 |
| miR-149-5p | 141.505 | 16.300 | 8.68 | 4.601E-03 |
| miR-548ah-5p | 45.720 | 6.100 | 7.50 | 4.601E-03 |
| miR-1285-5p | 36.365 | 5.660 | 6.42 | 4.601E-03 |
| miR-155-5p | 89.450 | 15.120 | 5.92 | 4.601E-03 |
| miR-3168 | 12.160 | 2.260 | 5.38 | 4.601E-03 |
| miR-575 | 17.580 | 1.600 | 10.99 | 5.939E-03 |
| miR-2682-5p | 88.895 | 9.880 | 9.00 | 5.939E-03 |
| miR-3613-3p | 19.670 | 2.200 | 8.94 | 5.939E-03 |
| miR-548y | 21.115 | 3.230 | 6.54 | 5.939E-03 |
| miR-378i | 53.340 | 9.960 | 5.36 | 5.939E-03 |
| miR-514b-5p | 52.870 | 12.240 | 4.32 | 5.939E-03 |
| miR-337-3p | 46.640 | 11.230 | 4.15 | 5.939E-03 |
| miR-598-3p | 13.085 | 3.500 | 3.74 | 5.939E-03 |
| miR-518b | 11.175 | 4.920 | 2.27 | 5.939E-03 |
| miR-499a-5p | 13.735 | 1.420 | 9.67 | 7.497E-03 |
| miR-486-3p | 43.995 | 6.160 | 7.14 | 7.512E-03 |
| miR-3065-5p | 220.830 | 35.810 | 6.17 | 7.512E-03 |
| miR-150-5p | 181.545 | 30.870 | 5.88 | 7.512E-03 |
| miR-1972 | 11.380 | 1.830 | 6.22 | 7.571E-03 |
| miR-548j-3p | 33.550 | 6.120 | 5.48 | 7.571E-03 |
| miR-548ai+miR-570-5p | 10.960 | 2.410 | 4.55 | 7.571E-03 |
| miR-548k | 13.205 | 1.000 | 13.21 | 8.119E-03 |
| miR-411-5p | 24.935 | 4.990 | 5.00 | 9.149E-03 |
| miR-196a-5p | 66.335 | 7.580 | 8.75 | 9.578E-03 |
| miR-526a+miR-518c-5p+miR-518d-5p | 17.760 | 2.700 | 6.58 | 9.578E-03 |
| miR-515-3p | 8.350 | 1.000 | 8.35 | 9.732E-03 |
| miR-563 | 11.345 | 1.420 | 7.99 | 1.003E-02 |
| miR-212-3p | 19.035 | 1.850 | 10.29 | 1.008E-02 |
| miR-188-5p | 9.255 | 1.710 | 5.41 | 1.008E-02 |
| miR-1305 | 11.965 | 2.610 | 4.58 | 1.008E-02 |
| miR-1293 | 5.085 | 1.000 | 5.09 | 1.090E-02 |
| miR-493-3p | 6.910 | 1.420 | 4.87 | 1.102E-02 |
| miR-4485-3p | 5.225 | 1.420 | 3.68 | 1.108E-02 |
| miR-574-5p | 143.340 | 24.890 | 5.76 | 1.110E-02 |
| miR-137 | 5.295 | 1.000 | 5.30 | 1.176E-02 |
| miR-873-3p | 23.595 | 3.180 | 7.42 | 1.199E-02 |
| miR-6721-5p | 9.665 | 1.580 | 6.12 | 1.199E-02 |
| miR-613 | 36.640 | 6.740 | 5.44 | 1.199E-02 |
| miR-656-3p | 24.845 | 7.150 | 3.47 | 1.199E-02 |
| miR-548v | 7.050 | 2.260 | 3.12 | 1.199E-02 |
| miR-4451 | 7.395 | 1.330 | 5.56 | 1.214E-02 |
| miR-302e | 10.430 | 1.790 | 5.83 | 1.220E-02 |
| miR-510-3p | 4.515 | 1.000 | 4.52 | 1.293E-02 |
| miR-640 | 14.325 | 1.420 | 10.09 | 1.341E-02 |
| miR-4443 | 10.675 | 1.500 | 7.12 | 1.471E-02 |
| miR-216a-5p | 11.920 | 1.500 | 7.95 | 1.490E-02 |
| miR-1185-2-3p | 14.350 | 1.920 | 7.47 | 1.490E-02 |
| miR-125b-5p | 43.345 | 8.590 | 5.05 | 1.490E-02 |
| miR-4536-5p | 55.945 | 11.770 | 4.75 | 1.490E-02 |
| miR-495-3p | 33.015 | 8.380 | 3.94 | 1.490E-02 |
| miR-1255a | 19.495 | 2.110 | 9.24 | 1.613E-02 |
| miR-612 | 125.630 | 15.540 | 8.08 | 1.613E-02 |
| miR-378h | 32.930 | 5.500 | 5.99 | 1.613E-02 |
| miR-1257 | 51.125 | 9.200 | 5.56 | 1.613E-02 |
| miR-1180-3p | 12.025 | 3.500 | 3.44 | 1.613E-02 |
| miR-200b-3p | 4.455 | 1.000 | 4.46 | 1.670E-02 |
| miR-1295a | 12.100 | 2.130 | 5.68 | 1.767E-02 |
| miR-140-3p | 9.170 | 2.240 | 4.09 | 1.767E-02 |
| miR-4536-3p | 8.300 | 1.000 | 8.30 | 1.826E-02 |
| miR-2053 | 8.115 | 1.000 | 8.12 | 1.829E-02 |
| miR-585-3p | 36.045 | 9.200 | 3.92 | 1.836E-02 |
| miR-489-3p | 8.270 | 3.110 | 2.66 | 1.836E-02 |
| miR-376a-3p | 66.530 | 31.990 | 2.08 | 1.836E-02 |
| miR-1183 | 7.625 | 1.420 | 5.37 | 1.906E-02 |
| miR-296-5p | 10.320 | 1.990 | 5.19 | 1.921E-02 |
| miR-3144-3p | 106.395 | 8.950 | 11.89 | 1.930E-02 |
| miR-128-1-5p | 39.255 | 7.180 | 5.47 | 1.933E-02 |
| miR-363-5p | 25.835 | 4.850 | 5.33 | 1.933E-02 |
| miR-519b-5p+miR-519c-5p+miR-523-5p+miR-518e-5p+miR-522-5p+miR-519a-5p | 9.260 | 1.790 | 5.17 | 1.933E-02 |
| miR-4531 | 83.930 | 17.250 | 4.87 | 1.933E-02 |
| miR-769-5p | 14.260 | 3.970 | 3.59 | 1.933E-02 |
| miR-301b-5p | 4.220 | 1.000 | 4.22 | 2.002E-02 |
| miR-181a-3p | 6.105 | 1.000 | 6.11 | 2.053E-02 |
| miR-1279 | 8.065 | 1.000 | 8.07 | 2.084E-02 |
| miR-937-3p | 6.445 | 1.290 | 5.00 | 2.103E-02 |
| miR-448 | 10.095 | 1.370 | 7.37 | 2.110E-02 |
| miR-362-3p | 3.440 | 1.000 | 3.44 | 2.193E-02 |
| miR-187-3p | 3.110 | 1.000 | 3.11 | 2.193E-02 |
| miR-644a | 41.590 | 12.510 | 3.32 | 2.247E-02 |
| miR-610 | 25.245 | 8.700 | 2.90 | 2.247E-02 |
| miR-151b | 4.290 | 1.170 | 3.67 | 2.270E-02 |
| miR-566 | 4.515 | 1.170 | 3.86 | 2.277E-02 |
| miR-511-5p | 10.675 | 1.420 | 7.52 | 2.293E-02 |
| miR-365a-3p+miR-365b-3p | 10.095 | 1.500 | 6.73 | 2.304E-02 |
| miR-324-3p | 6.450 | 1.420 | 4.54 | 2.304E-02 |
| miR-874-5p | 11.360 | 2.970 | 3.82 | 2.304E-02 |
| miR-548ad-3p | 18.075 | 1.920 | 9.41 | 2.307E-02 |
| miR-587 | 34.660 | 10.370 | 3.34 | 2.307E-02 |
| miR-371b-5p | 5.625 | 1.000 | 5.63 | 2.321E-02 |
| miR-3196 | 3.985 | 1.000 | 3.99 | 2.392E-02 |
| miR-381-5p | 9.330 | 1.000 | 9.33 | 2.448E-02 |
| miR-520g-3p | 6.225 | 1.000 | 6.23 | 2.448E-02 |
| miR-345-3p | 4.835 | 1.290 | 3.75 | 2.481E-02 |
| miR-454-3p | 7.555 | 2.610 | 2.89 | 2.481E-02 |
| miR-192-5p | 12.485 | 1.580 | 7.90 | 2.506E-02 |
| miR-625-5p | 25.910 | 8.240 | 3.14 | 2.513E-02 |
| miR-34a-5p | 4.570 | 1.850 | 2.47 | 2.516E-02 |
| miR-1266-5p | 7.085 | 1.000 | 7.09 | 2.531E-02 |
| miR-6724-5p | 5.345 | 1.000 | 5.35 | 2.613E-02 |
| miR-3202 | 7.605 | 1.000 | 7.61 | 2.666E-02 |
| miR-548m | 7.020 | 1.000 | 7.02 | 2.666E-02 |
| miR-326 | 4.870 | 1.000 | 4.87 | 2.666E-02 |
| miR-138-5p | 31.320 | 4.920 | 6.37 | 2.726E-02 |
| miR-1323 | 13.170 | 2.550 | 5.16 | 2.726E-02 |
| miR-1322 | 21.665 | 7.770 | 2.79 | 2.726E-02 |
| miR-548a-5p | 13.730 | 5.290 | 2.60 | 2.726E-02 |
| miR-301b-3p | 16.800 | 1.790 | 9.39 | 2.738E-02 |
| miR-628-5p | 8.300 | 1.350 | 6.15 | 2.738E-02 |
| miR-197-5p | 11.620 | 1.420 | 8.18 | 2.742E-02 |
| miR-206 | 7.605 | 2.260 | 3.37 | 2.742E-02 |
| miR-4488 | 6.610 | 2.110 | 3.13 | 2.742E-02 |
| miR-196a-3p | 4.160 | 1.000 | 4.16 | 2.757E-02 |
| miR-582-3p | 3.105 | 1.000 | 3.11 | 2.765E-02 |
| miR-1262 | 4.630 | 1.000 | 4.63 | 2.849E-02 |
| miR-509-5p | 6.840 | 1.080 | 6.33 | 2.945E-02 |
| miR-96-5p | 6.105 | 1.400 | 4.36 | 2.973E-02 |
| miR-551b-3p | 5.680 | 1.500 | 3.79 | 2.973E-02 |
| miR-2117 | 10.855 | 2.610 | 4.16 | 2.981E-02 |
| miR-518c-3p | 5.655 | 1.000 | 5.66 | 3.009E-02 |
| miR-607 | 5.155 | 1.000 | 5.16 | 3.009E-02 |
| miR-431-5p | 4.515 | 1.000 | 4.52 | 3.009E-02 |
| miR-532-3p | 3.910 | 1.000 | 3.91 | 3.009E-02 |
| miR-299-5p | 3.050 | 1.000 | 3.05 | 3.009E-02 |
| miR-507 | 2.910 | 1.000 | 2.91 | 3.009E-02 |
| miR-3690 | 5.225 | 1.000 | 5.23 | 3.098E-02 |
| miR-1973 | 6.305 | 1.290 | 4.89 | 3.208E-02 |
| miR-195-5p | 7.625 | 2.080 | 3.67 | 3.238E-02 |
| miR-152-3p | 5.225 | 1.420 | 3.68 | 3.242E-02 |
| miR-25-5p | 8.380 | 1.000 | 8.38 | 3.281E-02 |
| miR-133a-5p | 7.020 | 1.000 | 7.02 | 3.281E-02 |
| miR-548d-5p | 6.010 | 1.000 | 6.01 | 3.281E-02 |
| miR-1908-3p | 5.315 | 1.000 | 5.32 | 3.281E-02 |
| miR-505-3p | 4.480 | 1.000 | 4.48 | 3.281E-02 |
| miR-371a-5p | 3.910 | 1.000 | 3.91 | 3.281E-02 |
| miR-5196-5p | 8.940 | 1.990 | 4.49 | 3.289E-02 |
| miR-601 | 11.125 | 3.600 | 3.09 | 3.289E-02 |
| miR-548ar-3p | 4.800 | 1.000 | 4.80 | 3.374E-02 |
| miR-10a-5p | 9.560 | 1.420 | 6.73 | 3.442E-02 |
| miR-499a-3p | 6.035 | 1.000 | 6.04 | 3.442E-02 |
| miR-1287-3p | 3.775 | 1.000 | 3.78 | 3.442E-02 |
| miR-561-5p | 5.155 | 1.420 | 3.63 | 3.514E-02 |
| miR-592 | 13.045 | 1.830 | 7.13 | 3.523E-02 |
| miR-520a-5p | 7.285 | 1.790 | 4.07 | 3.523E-02 |
| miR-664b-3p | 4.300 | 1.000 | 4.30 | 3.563E-02 |
| miR-330-3p | 3.955 | 1.000 | 3.96 | 3.563E-02 |
| miR-450b-3p | 3.910 | 1.000 | 3.91 | 3.563E-02 |
| miR-208a-3p | 2.875 | 1.000 | 2.88 | 3.563E-02 |
| miR-553 | 7.890 | 1.080 | 7.31 | 3.733E-02 |
| miR-654-5p | 6.480 | 1.080 | 6.00 | 3.733E-02 |
| miR-369-3p | 4.825 | 1.000 | 4.83 | 3.733E-02 |
| miR-642a-3p | 6.010 | 1.330 | 4.52 | 3.733E-02 |
| miR-506-3p | 5.085 | 1.290 | 3.94 | 3.786E-02 |
| miR-1307-5p | 4.220 | 1.330 | 3.17 | 3.786E-02 |
| miR-219a-5p | 7.835 | 1.420 | 5.52 | 3.809E-02 |
| miR-3161 | 28.405 | 8.670 | 3.28 | 3.824E-02 |
| miR-483-3p | 6.985 | 2.260 | 3.09 | 3.824E-02 |
| miR-873-5p | 7.545 | 2.550 | 2.96 | 3.829E-02 |
| miR-338-5p | 3.495 | 1.000 | 3.50 | 3.867E-02 |
| miR-614 | 2.875 | 1.000 | 2.88 | 3.867E-02 |
| miR-190a-5p | 5.600 | 1.000 | 5.60 | 3.877E-02 |
| miR-329-3p | 5.225 | 1.000 | 5.23 | 3.877E-02 |
| miR-1304-5p | 5.155 | 1.000 | 5.16 | 3.877E-02 |
| miR-103a-3p | 4.915 | 1.000 | 4.92 | 3.877E-02 |
| miR-620 | 4.300 | 1.000 | 4.30 | 3.877E-02 |
| miR-548g-3p | 50.095 | 12.050 | 4.16 | 3.938E-02 |
| miR-499b-3p | 4.705 | 1.000 | 4.71 | 3.971E-02 |
| miR-939-5p | 7.040 | 1.000 | 7.04 | 3.981E-02 |
| miR-514a-5p | 6.875 | 1.000 | 6.88 | 3.981E-02 |
| miR-455-5p | 2.910 | 1.000 | 2.91 | 3.981E-02 |
| miR-651-5p | 7.580 | 1.000 | 7.58 | 4.056E-02 |
| miR-551a | 6.985 | 1.350 | 5.17 | 4.056E-02 |
| miR-141-3p | 7.495 | 1.420 | 5.28 | 4.101E-02 |
| miR-660-5p | 6.445 | 1.080 | 5.97 | 4.126E-02 |
| miR-199b-5p | 3.400 | 1.000 | 3.40 | 4.202E-02 |
| miR-548e-3p | 2.875 | 1.000 | 2.88 | 4.202E-02 |
| miR-203a-5p | 7.170 | 1.000 | 7.17 | 4.312E-02 |
| miR-153-3p | 4.175 | 1.000 | 4.18 | 4.312E-02 |
| miR-99a-5p | 5.225 | 1.000 | 5.23 | 4.391E-02 |
| miR-936 | 4.645 | 1.000 | 4.65 | 4.403E-02 |
| miR-10b-5p | 4.480 | 1.000 | 4.48 | 4.403E-02 |
| miR-595 | 3.510 | 1.000 | 3.51 | 4.403E-02 |
| miR-1827 | 6.955 | 1.420 | 4.90 | 4.449E-02 |
| miR-4421 | 18.715 | 1.420 | 13.18 | 4.476E-02 |
| miR-522-3p | 7.470 | 2.080 | 3.59 | 4.476E-02 |
| miR-494-3p | 1261.795 | 327.560 | 3.85 | 4.497E-02 |
| miR-302a-5p | 11.620 | 3.540 | 3.28 | 4.497E-02 |
| miR-1283 | 1260.285 | 396.930 | 3.18 | 4.497E-02 |
| miR-30e-5p | 129.990 | 46.230 | 2.81 | 4.497E-02 |
| miR-4647 | 3.035 | 1.000 | 3.04 | 4.562E-02 |
| miR-2110 | 4.525 | 1.000 | 4.53 | 4.667E-02 |
| miR-922 | 9.625 | 1.000 | 9.63 | 4.678E-02 |
| miR-100-5p | 8.050 | 1.000 | 8.05 | 4.678E-02 |
| miR-92b-3p | 5.860 | 1.000 | 5.86 | 4.678E-02 |
| miR-3615 | 4.880 | 1.000 | 4.88 | 4.678E-02 |
| miR-519c-3p | 4.835 | 1.000 | 4.84 | 4.678E-02 |
| miR-491-5p | 4.215 | 1.000 | 4.22 | 4.678E-02 |
| miR-577 | 8.300 | 2.550 | 3.25 | 4.688E-02 |
| miR-891a-5p | 4.915 | 1.000 | 4.92 | 4.761E-02 |
| miR-33b-5p | 3.775 | 1.000 | 3.78 | 4.761E-02 |
| miR-641 | 4.915 | 1.370 | 3.59 | 4.761E-02 |
| miR-501-3p | 5.060 | 1.000 | 5.06 | 4.768E-02 |
| miR-515-5p | 12.225 | 1.000 | 12.23 | 4.780E-02 |
| miR-514a-3p | 5.395 | 1.000 | 5.40 | 4.780E-02 |
| miR-193b-3p | 5.245 | 1.000 | 5.25 | 4.780E-02 |
| miR-345-5p | 4.515 | 1.000 | 4.52 | 4.780E-02 |
| miR-591 | 4.300 | 1.000 | 4.30 | 4.780E-02 |
| miR-671-3p | 6.125 | 1.350 | 4.54 | 4.811E-02 |
| miR-3195 | 5.250 | 1.420 | 3.70 | 4.811E-02 |
| miR-548al | 9.080 | 1.400 | 6.49 | 4.816E-02 |
| miR-626 | 8.020 | 1.330 | 6.03 | 4.816E-02 |
| miR-339-5p | 6.745 | 1.420 | 4.75 | 4.816E-02 |
| miR-802 | 9.015 | 1.920 | 4.70 | 4.844E-02 |
| miR-132-3p | 6.010 | 1.600 | 3.76 | 4.850E-02 |
| miR-1298-5p | 5.200 | 1.600 | 3.25 | 4.850E-02 |
| miR-518f-3p | 6.185 | 1.420 | 4.36 | 4.861E-02 |
| miR-504-5p | 12.645 | 1.920 | 6.59 | 4.866E-02 |
| miR-649 | 2.955 | 1.000 | 2.96 | 4.935E-02 |
| miR-936 | 4.645 | 1 | 4.65 | 0.044032 |
| miR-937-3p | 6.445 | 1.29 | 5.00 | 0.021032 |
| miR-939-5p | 7.04 | 1 | 7.04 | 0.03981 |
| miR-96-5p | 6.105 | 1.4 | 4.36 | 0.029732 |
| miR-99a-5p | 5.225 | 1 | 5.23 | 0.043911 |
| ***Under-expressed miRNAs*** |  |  |  |  |
| miR-199a-5p | 11.840 | 36.850 | 0.32 | 2.247E-02 |
| miR-142-3p | 64.745 | 129.960 | 0.50 | 2.307E-02 |
| miR-340-5p | 11.710 | 31.730 | 0.37 | 3.242E-02 |
| miR-15b-5p | 26.350 | 122.270 | 0.22 | 3.247E-02 |
| miR-15a-5p | 10.095 | 23.850 | 0.42 | 3.247E-02 |

*Note that a few miRNAs are combined in the same probe in the Nanostring assay.
