# Supplementary material for: Circulating miR-16-5p, miR-92a-3p, and miR-451a in Plasma from Lung Cancer Patients: Potential Application in Early Detection and a Regulatory Role in Tumorigenesis Pathways
Source: Cancers (Basel). 2020 Jul 27;12(8):2071. doi: 10.3390/cancers12082071 (PMC7465670; doi:10.3390/cancers12082071)
Supplement: Supplementary file 1 [file cancers-12-02071-s001.zip › Table S4.docx]

**Table S4.** List of the 216 commonly over-expressed miRNAs in plasma from LUAD and LUSC patients.

| **miRNAs** |  |  |
| --- | --- | --- |
| miR-16-5p | miR-510-3p | miR-1257 |
| miR-451a | miR-137 | miR-4536-5p |
| miR-92a-3p | miR-362-3p | miR-514b-5p |
| miR-25-3p | miR-577 | miR-1322 |
| miR-494-3p | miR-486-3p | miR-654-5p |
| miR-1285-5p | miR-100-5p | miR-455-5p |
| miR-125b-5p | miR-381-5p | miR-651-5p |
| miR-448 | miR-939-5p | miR-922 |
| miR-155-5p | miR-499b-3p | miR-1287-3p |
| miR-2682-5p | miR-499a-5p | miR-212-3p |
| miR-548j-3p | miR-1972 | miR-874-5p |
| miR-378i | miR-587 | miR-188-5p |
| miR-574-5p | miR-607 | miR-299-5p |
| miR-873-3p | miR-450b-3p | miR-301b-5p |
| miR-197-5p | miR-1304-5p | miR-25-5p |
| miR-216a-5p | miR-196a-3p | miR-532-3p |
| miR-548al | miR-4536-3p | miR-553 |
| miR-3065-5p | miR-200b-3p | miR-1185-2-3p |
| miR-149-5p | miR-507 | miR-644a |
| miR-625-5p | miR-582-3p | miR-548v |
| miR-612 | miR-614 | miR-613 |
| miR-1323 | miR-103a-3p | miR-585-3p |
| miR-150-5p | miR-338-5p | miR-489-3p |
| miR-1283 | miR-936 | miR-363-5p |
| miR-1180-3p | miR-548ad-3p | miR-1293 |
| miR-133a-5p | miR-302a-5p | miR-891a-5p |
| miR-515-5p | miR-2117 | miR-330-3p |
| miR-501-3p | miR-548g-3p | miR-187-3p |
| miR-566 | miR-514a-5p | miR-595 |
| miR-296-5p | miR-199b-5p | miR-664b-3p |
| miR-1908-3p | miR-509-5p | miR-371b-5p |
| miR-6724-5p | miR-2110 | miR-4451 |
| miR-505-3p | miR-551b-3p | miR-551a |
| miR-153-3p | miR-192-5p | miR-601 |
| miR-1307-5p | miR-626 | miR-548ar-3p |
| miR-3144-3p | miR-10a-5p | miR-640 |
| miR-628-5p | miR-1305 | miR-324-3p |
| miR-378h | miR-563 | miR-141-3p |
| miR-337-3p | miR-151b | miR-506-3p |
| miR-3613-3p | miR-620 | miR-561-5p |
| miR-610 | miR-345-5p | miR-1255a |
| miR-30e-5p | miR-548d-5p | miR-345-3p |
| miR-548y | miR-518c-3p | miR-519c-3p |
| miR-3168 | miR-548e-3p | miR-365a-3p+miR-365b-3p |
| miR-548k | miR-193b-3p | miR-873-5p |
| miR-92b-3p | miR-208a-3p | miR-656-3p |
| miR-4443 | miR-329-3p | miR-495-3p |
| miR-301b-3p | miR-371a-5p | miR-206 |
| miR-196a-5p | miR-431-5p | miR-937-3p |
| miR-526a+miR-518c-5p+  miR-518d-5p | miR-4531 | miR-369-3p |
| miR-548ah-5p | miR-1295a | miR-1298-5p |
| miR-411-5p | miR-190a-5p | miR-138-5p |
| miR-3161 | miR-642a-3p | miR-1183 |
| miR-195-5p | miR-575 | miR-1827 |
| miR-1266-5p | miR-499a-3p | miR-548a-5p |
| miR-514a-3p | miR-326 | miR-518f-3p |
| miR-3690 | miR-3202 | miR-641 |
| miR-203a-5p | miR-520g-3p | miR-4485-3p |
| miR-99a-5p | miR-181a-3p | miR-4421 |
| miR-1973 | miR-649 | miR-592 |
| miR-511-5p | miR-96-5p | miR-491-5p |
| miR-515-3p | miR-6721-5p | miR-302e |
| miR-591 | miR-3615 | miR-802 |
| miR-3196 | miR-548m | miR-140-3p |
| miR-2053 | miR-33b-5p | miR-519b-5p+miR-519c-5p+miR-523-5p+miR-518e-5p+miR-522-5p+miR-519a-5p |
| miR-4647 | miR-1279 | miR-548ai+miR-570-5p |
| miR-10b-5p | miR-1262 | miR-520a-5p |
| miR-128-1-5p | miR-219a-5p | miR-660-5p |
| miR-522-3p | miR-4488 | miR-152-3p |

*Note that a few miRNAs are combined in the same probe in the Nanostring assay. Expression levels and associated *p*-values are shown in Supplementary Tables 2 and 3, respectively, for LUAD and LUSC patients, compared to healthy controls.
