# Supplementary material for: Circulating miR-16-5p, miR-92a-3p, and miR-451a in Plasma from Lung Cancer Patients: Potential Application in Early Detection and a Regulatory Role in Tumorigenesis Pathways
Source: Cancers (Basel). 2020 Jul 27;12(8):2071. doi: 10.3390/cancers12082071 (PMC7465670; doi:10.3390/cancers12082071)
Supplement: Supplementary file 1 [file cancers-12-02071-s001.zip › Table S8_Table S9_Table S10.docx]

**Table S8.** Demographic and histopathological data of patients.

| **Variables** | **Discovery**  **(n=38)** | **Validation**  **(n=40)** |
| --- | --- | --- |
| **Age (years)**  Mean (SD) | 66 (11) | 68 (11) |
| **Sex** |  |  |
| Female | 18 | 20 |
| Male | 20 | 20 |
| **Smoking** |  |  |
| Yes | 38 | 40 |
| **Histological subtype** |  |  |
| Adenocarcinoma | 22 | 18 |
| Squamous cell carcinoma | 16 | 22 |
| **Disease stage** |  |  |
| IA | 12 | 14 |
| IB | 10 | 12 |
| IIA | 2 | 4 |
| IIB | 5 | 8 |
| IIIA | 5 | 1 |
| IIIB | 2 | - |
| IV | 2 | 1 |

There were no statistical differences between the discovery and validation sets.

Disease Stage: according to the 8^th^ edition Lung Cancer TNM Staging.

**Table S9.** Oligonucleotide sequences of exogenous miRNAs used as spike-in controls.

| **Exogenous**  **miRNA** | **miRBase**  **accession #** | **Sequence** |
| --- | --- | --- |
| ath-miR-159a | MIMAT0000177 | 5'-UUUGGAUUGAAGGGAGCUCUA-3' |
| cel-miR-248 | MIMAT0000304 | 5'-AUACACGUGCACGGAUAACGCUCA-3' |

ath-miR-159: *Arabidopsis thaliana* miR-159; cel-miR-248: *Caenorhabditis elegans* miR-248.

**Table S10.** TaqMan® primer sequences used for validation experiments.

| **miRNA ID** | **Assay ID** | **Primer sequence** |
| --- | --- | --- |
| miR-16-5p | 477860 | UAGCAGCACGUAAAUAUUGGCG |
| miR-92a-3p | 000431 | UAUUGCACUUGUCCCGGCCUGU |
| miR-451a | 001105 | AAACCGUUACCAUUACUGAGUUU |
| miR-106b-5p | 478412 | UAAAGUGCUGACAGUGCAGAU |
| miR-155-5p | 477927 | UUAAUGCUAAUCGUGAUAGGGGU |
| miR-217 | 478773 | UACUGCAUCAGGAACUGAUUGGA |
| miR-1285-3p | 478687 | UCUGGGCAACAAAGUGAGACCU |
| miR-1285-5p | 479565 | GAUCUCACUUUGUUGCCCAGG |
| miR-148b-3p | 477824 | UCAGUGCAUCACAGAACUUUGU |
| miR-378e | 478537 | ACUGGACUUGGAGUCAGGA |
| miR-484 | 478308 | UCAGGCUCAGUCCCCUCCCGAU |
| miR-664a-3p | 478193 | UAUUCAUUUAUCCCCAGCCUACA |
