# Supplementary figures and images for: Circulating miR-16-5p, miR-92a-3p, and miR-451a in Plasma from Lung Cancer Patients: Potential Application in Early Detection and a Regulatory Role in Tumorigenesis Pathways
Source: Cancers (Basel). 2020 Jul 27;12(8):2071. doi: 10.3390/cancers12082071 (PMC7465670; doi:10.3390/cancers12082071)

Normalized by miRNA 93

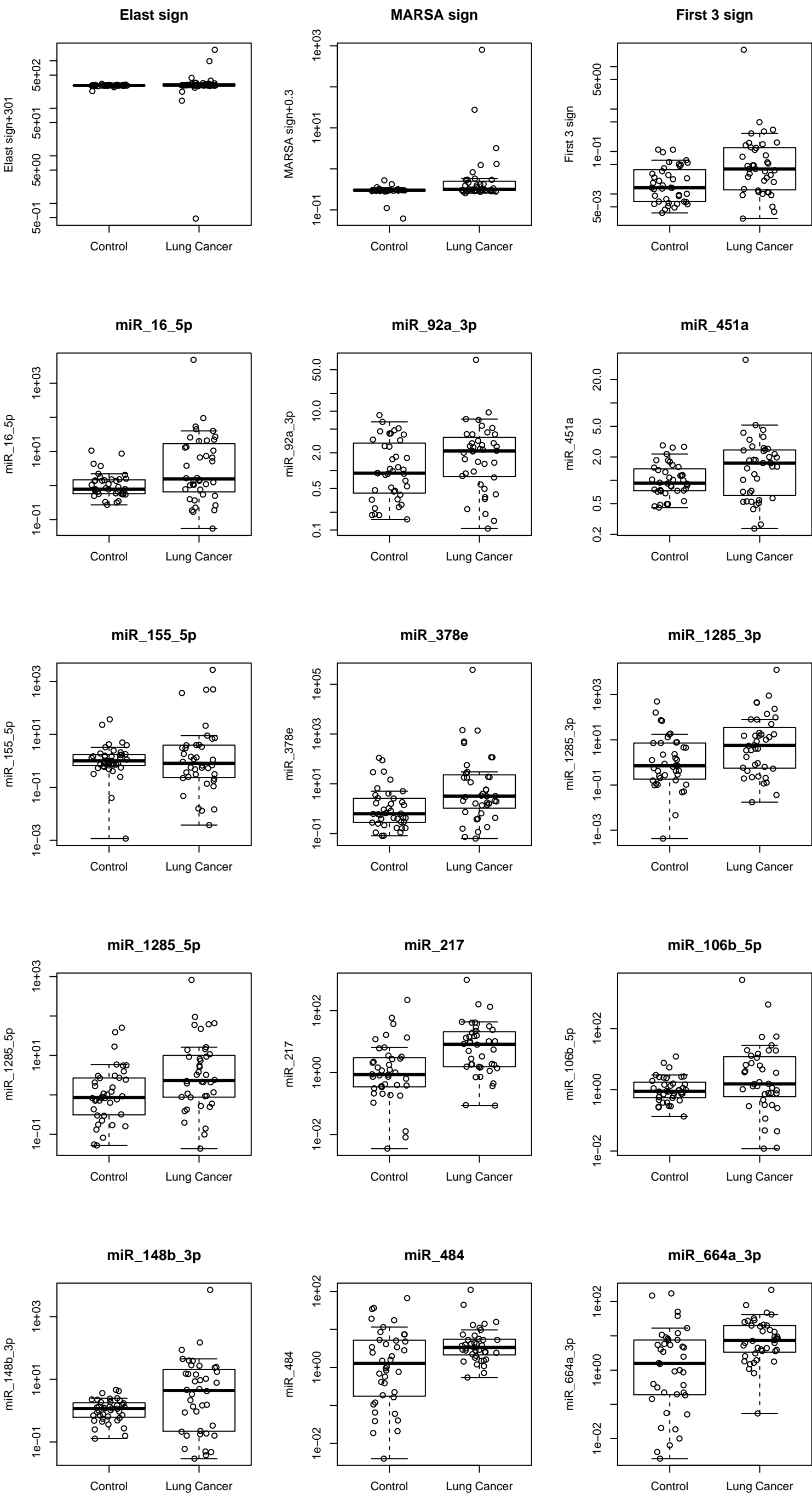

Supplement: Supplementary file 1 [file cancers-12-02071-s001.zip › Figure S1.pdf]

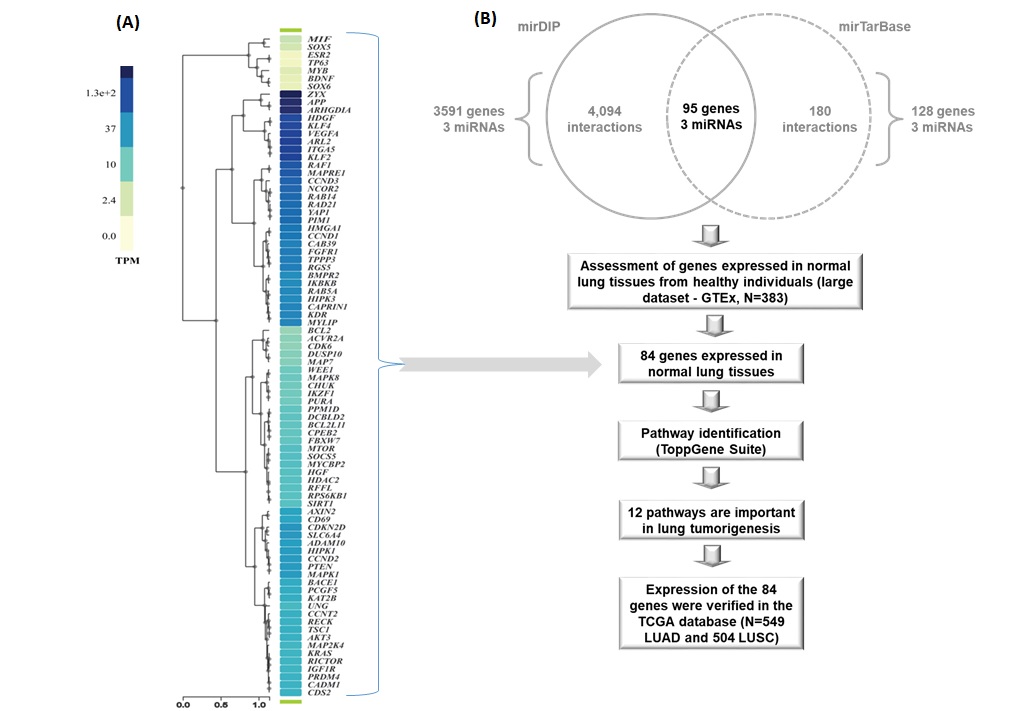

Supplement: Supplementary file 1 [file cancers-12-02071-s001.zip › Figure S2.jpg]
